# Innovation in Pain Rehabilitation Using Co-Design Methods During the Development of a Relapse Prevention Intervention: Case Study

**DOI:** 10.2196/18462

**Published:** 2021-01-20

**Authors:** Stefan Elbers, Christa van Gessel, Reint Jan Renes, Remko van der Lugt, Harriët Wittink, Sander Hermsen

**Affiliations:** 1 Research Group Lifestyle and Health Utrecht University of Applied Sciences Utrecht Netherlands; 2 Department of Rehabilitation Medicine Faculty of Health, Life Sciences and Medicine Maastricht University Maastricht Netherlands; 3 Co-design Research Group Utrecht University of Applied Sciences Utrecht Netherlands; 4 Research Group Psychology of Sustainable Cities Amsterdam University of Applied Sciences Amsterdam Netherlands; 5 OnePlanet Research Center imec The Netherlands Wageningen Netherlands

**Keywords:** co-design, participatory design, chronic pain, intervention development, rehabilitation, behavior change, relapse, prevention

## Abstract

**Background:**

Many intervention development projects fail to bridge the gap from basic research to clinical practice. Instead of theory-based approaches to intervention development, co-design prioritizes the end users’ perspective as well as continuous collaboration between stakeholders, designers, and researchers throughout the project. This alternative approach to the development of interventions is expected to promote the adaptation to existing treatment activities and to be responsive to the requirements of end users.

**Objective:**

The first objective was to provide an overview of all activities that were employed during the course of a research project to develop a relapse prevention intervention for interdisciplinary pain treatment programs. The second objective was to examine how co-design may contribute to stakeholder involvement, generation of relevant insights and ideas, and incorporation of stakeholder input into the intervention design.

**Methods:**

We performed an embedded single case study and used the double diamond model to describe the process of intervention development. Using all available data sources, we also performed deductive content analysis to reflect on this process.

**Results:**

By critically reviewing the value and function of a co-design project with respect to idea generation, stakeholder involvement, and incorporation of stakeholder input into the intervention design, we demonstrated how co-design shaped the transition from ideas, via concepts, to a prototype for a relapse prevention intervention.

**Conclusions:**

Structural use of co-design throughout the project resulted in many different participating stakeholders and stimulating design activities. As a consequence, the majority of the components of the final prototype can be traced back to the information that stakeholders provided during the project. Although this illustrates how co-design facilitates the integration of contextual information into the intervention design, further experimental testing is required to evaluate to what extent this approach ultimately leads to improved usability as well as patient outcomes in the context of clinical practice.

## Introduction

Only a fraction of intervention development projects is able to bridge the translational gap from scientific research to clinical practice [1-4]. An important factor for this limited uptake may be that contextual factors, such as stakeholder acceptability or implementation in existing practices, receive little attention during earlier development stages [5]. For example, many intervention development guidelines emphasize the formulation of an underlying theoretical construct and subsequent experimental testing to validate each assumed causal step, but only address implementation and feasibility after the intervention development phase has been completed [6-8]. Consequently, theoretically sound interventions may be discarded due to insufficient attention to crucial translational factors such as low perceived utility by patients or health care providers (HCPs), inconvenient navigation, or a discrepancy between the intervention requirements and patients’ preferences [9-14].

An opportunity to increase the emphasis on these factors is to incorporate co-design methods. Co-design not only is characterized by an incremental knowledge over multiple development cycles [15] but also specifically emphasizes empathizing with each stakeholder, integrating conflicting requirements, and quickly transitioning ideas to testable prototypes. Co-design differs from other design methodologies in that it involves a range of tools and exercises to optimize collaboration between professional designers and people who are not trained in the design process, such as patients and therapists [16]. Done rightly, co-design brings together different views, input, and competencies of people with a variety of perspectives to address a specific problem [17,18]. As a result, this approach should increase the acceptability and integration of the intervention in existing clinical practice by accommodating relevant contextual factors that have been identified by stakeholders in the development process.

Although co-design is increasingly adopted in the development of health care interventions (eg, [18-23]), prior studies have indicated that effective co-design is not without its challenges. For example, the process of engaging all stakeholders can be time-consuming and intensive. This can be particularly difficult in the context of health care because HCPs generally have a high workload [20], and participating patients often do not directly benefit from the development projects, which could negatively influence their motivation and engagement. Moreover, patients, policy makers, and HCPs can experience conflicting interests during intervention development projects, because the assumed best possible care is generally limited by finite resources or specific treatment guidelines within a particular health care environment [24]. Factors such as these could endanger the main principles of co-design and should be further examined in the context of health care [18,19].

### Co-Design in the Context of Chronic Pain

In the present project, called the SOLACE project (grant number: SIA RAAK 2014-01-23), we developed a relapse prevention intervention for patients with chronic musculoskeletal pain who participate in an interdisciplinary, multimodal pain treatment program. The primary reason for adopting a co-design approach was that, despite high prevalence rates of relapse after successful treatment, there is a paucity of available research to explain relapse for this particular population [25,26]. In these situations, a design-based approach may be particularly appropriate, because it allows for new insights to be recursively fed into future development cycles, thereby gradually increasing the knowledge base over time [15,27]. Because patients with chronic pain often experience distrust from their personal and medical environment [28], co-design may also prove effective in empowering patients to participate in the development process and to actively share their opinions and ideas [23,29].

### Objectives

To increase understanding of how co-design can be successfully applied in the development of interventions in the health care domain, more examples of good practice are needed [13,18,30]. Therefore, our research question was to what extent co-design practices facilitate the translation of meaningful stakeholder experiences into the design of a health care intervention. Our first aim was to provide a detailed overview of all co-design activities that were employed during the course of an example project. Our second aim was to reflect on this overview and examine how co-design may contribute to stakeholder involvement, generation of relevant insights and ideas, and incorporation of stakeholder input into the intervention design. We hypothesized that co-design activities would facilitate the generation of relevant experiences and insights from stakeholders and stimulate their active participation during this project. Consequently, we expected that this would yield prototypes that were aligned with clinical practice and would resonate with end users.

## Methods

### Design

We performed an embedded single case study [31], in which we analyzed and evaluated all co-design activities that were related to the development process of the SOLACE project. Throughout the study, the researchers followed a participatory action research (PAR) approach, which is characterized by active collaboration with the people of interest, rather than only researching them. PAR also emphasizes respectful cooperation between stakeholders and researchers including collective decision making and a bidirectional transfer of knowledge over multiple iterative development cycles [32-34], which is in accordance with co-design methods [35]. During each cycle, insight is acquired through action (eg, by letting patients interact with preliminary prototypes), empowerment of stakeholders (eg, by patient involvement in co-design sessions), and subsequent reflection [32].

### Participants

The SOLACE project consortium consisted of 2 interdisciplinary multimodal pain treatment centers, the Royal Dutch Society for Physical Therapy, The Dutch National Pain Patient Advocacy Organisation, and 4 research groups with a respective interest in chronic pain treatment (2 groups), co-design, and behavior change. All consortium partners assisted with the recruitment of participants when this was required at specific co-design activities, including patients and their spouses, HCPs, designers, researchers, and students. The core team was composed of 3 researchers, each from a different research group. This team was responsible for the planning and preparation of the co-design activities. To monitor overall progress, a steering committee was formed, which included representatives of all consortium partners. Ethical approval for this study was granted by the local ethics committee (Medical Research Ethics Committee Atrium 15-N-120).

### Materials

#### Co-Design Methods

In interviews and co-creation sessions, the core team adopted various co-design methods, including generative techniques, contextual interviews, system mapping, and prototyping. These methods were adopted to facilitate stakeholder participation during key moments in the design process: generative techniques to elicit tacit knowledge and latent needs, contextual interviews to increase empathy, system mapping to develop a comprehensive overview of the acquired data, and prototyping to make ideas tangible and possible to experience. Co-creation sessions included multiple co-design methods and were specifically employed to empower a variety of stakeholders to participate in the design process.

#### Semistructured Contextual Interviews

At various time points in the project, we interviewed patients and HCPs. The interviews were performed by 2 researchers and were conducted in the everyday context of the HCPs (treatment facility) and patients (at home). To activate prior knowledge and experiences, all participants received “sensitizers”—assignments that stimulated thinking about relevant topics—before the interview (see page 4 in Multimedia Appendix 1) [36]. During the interview, the primary interviewer explored participants’ beliefs, needs, and experiences using open questions and various generative techniques. The second interviewer took notes and asked additional questions to ensure that all topics that the research team identified during preparatory sessions were covered. Data were collected by audio recording and note taking. Directly after the interview, both interviewers discussed their impressions and updated their notes.

#### Generative Techniques

To explore participants’ ideas, needs, and values beyond their first response, various generative techniques were employed during interview and design sessions. These techniques aim to bring up “tacit knowledge” by addressing social, emotional, and functional elements related to a topic of interest [36]. For example, to promote a more personal acquaintance during the interview sessions, participants were asked to introduce themselves by selecting 3 pictures from a deck of cards illustrated with ambiguous images that symbolized their personal values. The core team also used journey mapping during interviews (see page 3 in Multimedia Appendix 1). This technique enabled all attendees to collaboratively construct a graphic visualization or a timeline that illustrated their experiences with interdisciplinary multimodal pain treatment [37,38].

#### Prototyping and Provotyping

A key element of PAR is to increase insight by reflecting on actual interactions with prototypes. As Step 1.3 on page 5 of Multimedia Appendix 1 illustrates, the interaction with these objects stimulated the individual to envision future possibilities or to visualize concept ideas. The process of prototyping allows participants to actively engage with objects that were based on preliminary outcomes and encompass operationalizations of the concept of interest [39]. Provotyping takes place with objects that are not directly related to the final result but are specifically designed to test a specific hypothesis or elicit a particular response [40].

#### System Maps and Personas

System mapping is a method for creating a visual representation of interacting variables that facilitates the understanding of complex systems [41]. System maps typically include a framework of interrelated components, as well as clarifying examples of quotes and pictures. These maps can be presented in posters or cards and are useful to share data to stakeholders in an accessible way (see pages 7-9 in Multimedia Appendix 1). Moreover, it provides participants with the opportunity to jointly reflect on the data and update ideas during co-creation sessions.

A specific way to represent the data as a coherent “whole” for usage throughout co-design activities is by creating personas: fictitious archetypes of users, each reflecting a distinct pattern in goals, attitudes, and behaviors based on empirical research among potential users. With personas, it is possible to highlight certain areas of tension or to facilitate discussion of important patient characteristics [42].

#### Co-Creation Sessions

We used co-creation sessions at key moments during the project to discuss and reflect on the collected data, to generate new ideas, and to make decisions regarding future development directions (see page 10 in Multimedia Appendix 1). A typical session would take 4 hours and involved 10-20 stakeholders. The core team prepared the sessions by formulating desired outcomes and setting up system maps to present the data. During the sessions, 2 designers operated as workshop facilitators and used various assignments (eg, prioritizing ideas for prototype concepts) to work towards the desired outcomes in an open atmosphere where everyone was invited to actively participate. All written session data (eg, posters, drawings, notes) were collected and discussed during core team evaluation meetings directly after the session. To maintain involvement and commitment between the sessions, the core team sent bimonthly newsletters and posted updates on the project websites.

### Measurements and Analysis

#### Dataset

The dataset for this case study consisted of 4 different sources. To capture the results of the design methods, researchers documented each design and research activity, using observation notes, pictures, audio files, or video clips. In addition to the session documentation, researchers also organized reflective sessions directly after a co-design activity to summarize the output of co-creation sessions (eg, notes or post-its) into system maps. These maps served both as a descriptive analysis of the data as well as for input during subsequent co-design sessions. The dataset also consisted of minutiae of steering committee meetings and a retrospective project journey. This journey was the result of a reviewing session, where researchers and members of the steering committee chronologically described and discussed critical incidents.

#### Data Analysis

**Figure 1:**
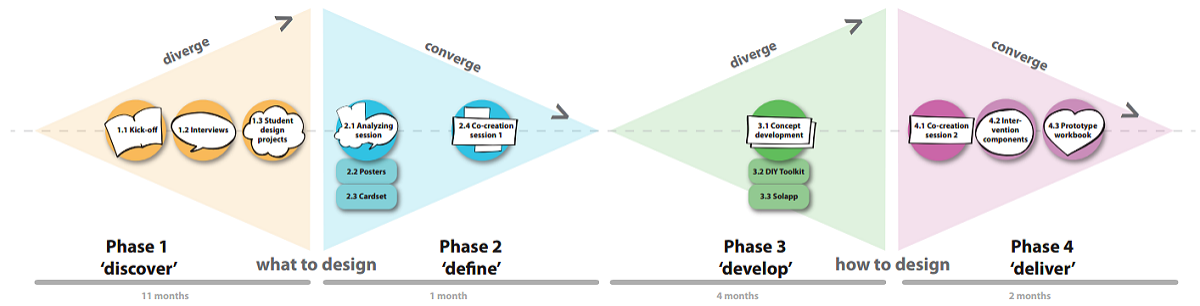
Overview of the co-design development process.

We used a deductive content approach to identify information within the dataset that relates to our main themes: stakeholder involvement, generation of insights, and incorporation of stakeholder input. We defined stakeholder involvement as the commitment to participate in the development project, to collaborate with other stakeholders during design activities, and to actively participate during these sessions. Generation of insights referred to the extent by which co-design activities resulted in an increased understanding of the problem of interest that could inform subsequent development activities. Incorporation of stakeholder needs was defined as the extent by which prototypes were based on stakeholder perceptions, judgments, and evaluations from co-design activities. Furthermore, we adopted the Double Diamond model to describe the design process along 4 development stages [43]. The Double Diamond model contains 2 sequences of diverging and converging (Figure 1). In diverging phases, choice options and discrepancies are created; in the converging phases, these ideas are refined and considered to make design choices with respect to the prototype. In the first diamond, the “Discover” (diverging) and “Define” (converging) phases relate to acquiring insights on *what* to design. In the second diamond, the “Develop” and “Deliver” relate to further exploring the ideas on *how* to optimally design the final concepts. To illustrate how co-design contributed to the intervention development at each phase, we combined all data sources to provide both a descriptive overview and an in-depth reflection with respect to our main themes. In addition, Multimedia Appendix 1 provides a chronological overview of the development process and includes examples of co-design methods, data segments, and pictures of co-design activities.

## Results

### Phase 1: “Discover’”

#### Description

In the “Discover” phase, we aimed to generate a deeper understanding of factors influencing relapse after successful rehabilitation. The primary activities took place over a period of 11 months and consisted of 3 kick-off sessions, 20 stakeholder interviews (12 HCPs, 8 patients), and a student design project. In the first kick-off session, representatives from all consortium partners were present to discuss the project planning and to decide how co-design would be implemented throughout the project. Representatives also participated in co-creation by using their professional and personal experiences to formulate initial ideas on relapse (see page 3 of Multimedia Appendix 1). These activities were repeated during introductory visits by the core team at the 2 participating pain treatment centers. During these visits, the core team also observed multiple treatment sessions and were given a detailed explanation about dose and content of the included treatment modalities. Subsequently, 20 semistructured interviews of approximately 1 hour were conducted and transcribed (see page 4 in Multimedia Appendix 1). During the final activity of phase 1, 60 students divided over 16 teams formulated hypotheses based on the previously collected data and designed provotypes to test their ideas with both healthy participants and patients with chronic pain (see page 5 of Multimedia Appendix 1). At the start of each week, they updated their provotypes based on the received feedback. During the final project session, all teams presented their final provotypes as well as their collected insights to members of the consortium.

#### Reflection

In phase 1, we were able to create a large qualitative dataset. This dataset not only contained experiences and ideas of stakeholders but also included specific feedback in response to multiple provotypes on a wide array of topics. The consecutive planning of the 3 key activities enabled us to iteratively expand our insights on relapse after pain treatment: Interviews were prepared by using the insights from the kick-off sessions, and the student teams could build upon the preliminary analysis of the available interview data. The participating stakeholders responded positively to the co-design approach and cooperated actively during the sessions and interviews. Despite their inexperience with co-design, the sessions were considered accessible, pleasant, and relevant. However, medical ethical screening procedures and personnel deployment planning limited the possibility for last-minute requests or invitations for including HCPs and patients. The obtained dataset of patient and HCP responses also contributed to a deeper understanding of relevant factors related to relapse, which provided a solid base for further intervention development. For example, the interviews revealed important contextual information such as a “feelings of emptiness after treatment,” “difficulties sharing treatment experiences with friends and family,” and “the different context between the rehabilitation center and the personal environment.”

### Phase 2: “Define”

#### Description

The “Define” phase lasted for 1 month and started with thematically organizing the interviews by means of open coding by the core team (see page 6 of Multimedia Appendix 1) [44,45]. This resulted in 8 main themes and 45 subthemes of factors associated with relapse after successful treatment (see page 7 in Multimedia Appendix 1). To facilitate subsequent co-design activities, the themes were rephrased as questions, plotted on posters, and illustrated with exemplary quotes and figures (see page 8 in Multimedia Appendix 1 for an example). In addition, the core team developed a set of 74 stimulus cards that were designed to facilitate the discussion of specific insights or principles [46]: 36 cards contained insights from the student project, 15 cards contained relevant theory on behavior regulation, and 23 cards contained theory related to chronic pain treatment (see page 9 in Multimedia Appendix 1). Subsequently, patients (4), HCPs (4), researchers (9), designers (6), and students (3) were invited for a co-creation session (see page 10 in Multimedia Appendix 1). During the first assignment, participants were asked to examine the posters and extend them with their own ideas or with stimulus cards. This resulted in 121 notes and 42 cards that were added to the posters. In the second assignment, subgroups were made of participants with varying backgrounds. Each group was instructed to select 1 theme and use the available information to develop an intervention concept. A professional draftsman supported the session by immediately visualizing intervention ideas. The final part of the session consisted of a plenary session where all 5 concepts were presented. During the subsequent discussion, the concepts were compared, and various overarching topics emerged, including “maintaining the positive development after treatment” and “reflection and self-monitoring.” In a subsequent meeting, the steering committee merged these overarching topics into 2 concept ideas: positive reinforcement and direct feedback. The “Define” phase concluded with a design briefing, where the core team commissioned 3 students to develop these ideas into tangible rudimentary prototypes as part of their graduation project.

#### Reflection

The final system map that included both posters and the card set provided a complete overview of the collected data. This presentation form stimulated participants to combine various insights to develop concept interventions. With respect to stakeholder involvement, the number of patients and HCPs was lower than originally planned. The duration of the session and traveling distance required participants to block a full day, which turned out to be difficult to organize. In line with our findings in phase 1, the co-design methods successfully engaged nonexperts in the design process. The assignment to create concept intervention ideas was concrete and tangible. The resulting 5 concepts were associated with earlier identified patient needs, were grounded in contextual information, and contained relevant insights on relapse prevention. For example, one concept idea focused on monitoring and recognizing early signals of relapse, which was based on stimulus cards (eg, a research insight related to difficulties in unbiased self-monitoring of behavior), interview data (eg, a quote from HCP on the possibility of daily feedback via eHealth), and newly added notes (eg, patient feedback should always be related to patient-specific goals). However, only a fraction of the possible combinations of cards and system maps was explored during this session. Limited time and resources prevented organizing additional sessions to cross-validate the results and achieve saturation.

### Phase 3: “Develop”

#### Description

During the 4 months of the “Develop” phase, students held 5 focus groups to regularly test their ideas with patients and HCPs (see page 11 in Multimedia Appendix 1). For example, by discussing the role of personal values within the treatment program, the students found supporting evidence that these values were strongly related to treatment goals, which subsequently guided the operationalization of the valued-based action plan in one of the rudimentary prototypes. Based on stakeholder feedback and weekly evaluation sessions with the core team, the students worked towards a final rudimentary prototype. One student focused on the transfer of important treatment insights to each patients’ personal context. She developed a toolbox that contained various methods to capture and store therapy insights in order to facilitate retrieval in a relevant personal context (eg, using a personal picture as memory cue for an important moment during treatment). The other 2 students focused on the generation of valued-based goals and the formulation of action plans for each consecutive step towards the goal. Their final rudimentary prototype consisted of a mockup mobile app, allowing participants to browse through all steps that were required to formulate and plan a valued-based goal. In Multimedia Appendix 1, pages 12-13 show the final versions of these rudimentary prototypes.

#### Reflection

This phase was characterized by a shift from “what” to “how” to design. Accordingly, presentation form, usability, and implementation into existing treatment practice became increasingly relevant. To engage stakeholders, the students visited the treatment centers on multiple occasions. In contrast to other phases, the patients and HCPs could provide feedback on ideas, but were not involved in the decision-making process regarding the final design of the rudimentary prototype, which potentially influenced their commitment. Moreover, their reduced involvement in this phase resulted in limited information regarding the applicability of the rudimentary prototypes in clinical practice.

### Phase 4: “Deliver”

#### Description

In the final phase, the core team merged both rudimentary prototypes into one final prototype intervention over a period of 2 months. To do so, the core team organized a final co-creation session, where the students presented their concepts. The aim of this session was to receive final feedback on the potential value and function of both rudimentary prototypes and to formulate a recommendation to the steering committee with respect to the final prototype design. To facilitate this process, stakeholders (n=14) were instructed to reflect on the concepts by taking various patient perspectives into account. For this purpose, 4 personas were created with variation on 2 characteristics that were often discussed during previous patient interviews. Each persona had either a high or low level of social support and a high or low tendency to protect personal boundaries. In Multimedia Appendix 1, page 14 depicts the discussions between stakeholders as well as the poster that explained the 4 personas. The final conclusion was that both rudimentary prototypes had potential as supportive treatment modalities to prevent relapse after successful treatment. Furthermore, future testing and development should primarily focus on optimizing the active treatment components and calibrating the intervention to the treatment program.

Based on this advice, the steering committee decided to merge both rudimentary prototypes into 1 prototype workbook. The core team composed a list of individual intervention components from each rudimentary prototype (eg, a prompt to set calendar reminders after a goal-setting procedure) and coded these according to the Behaviour Change Technique Taxonomy V1 (see page 15 in Multimedia Appendix 1) [47]. Subsequently, they determined how to transfer the components to a workbook version and performed literature searches to find ideas for optimizing the effectiveness of each component. For example, to assist the formulation of personal values, various value generation procedures were found (eg, [48]) and integrated into the prototype. In addition, the core team checked to which extent the list of intervention components corresponded with the themes of the interview dataset. Of the 19 intervention components, 17 components were related to one or more themes from the dataset, and 27 of the 45 themes were related to one or more intervention components. For example, 4 components in the goal-setting intervention, including specific probing questions to help formulate meaningful values, were associated with the theme “remembering important goals and values after treatment.” A designer, a text editor, and 3 HCPs provided feedback with the conversion to a paper workbook intervention and respectively focused on the design, readability, and appropriate terminology. In Multimedia Appendix 1, page 16 shows examples of the 2 included strategies: the value-based goal forms (b and c) and the Insight Cards (d).

#### Reflection

Previous difficulties with recruiting sufficient patients for co-creation sessions caused us to search for alternative ways to include their viewpoint. The personas proved a useful method to incorporate various patient perspectives by proxy during the evaluation of the rudimentary prototypes. Furthermore, the validation check indicated that the majority of the intervention components could be traced back to the original stakeholder themes from the interventions in the “Discover” phase and vice versa. This illustrates that stakeholder input has been incorporated in the design. However, the decision to combine both prototype ideas into one intervention was unexpected, which resulted in last-minute planning and consequently in limited stakeholder involvement during the design of the workbook. This may threaten the usability of this prototype in clinical practice.

## Discussion

### Principal Findings

The primary aim of this study was to reflect on the value and function of co-design methodology during the development of an intervention that prevents relapse after successful pain treatment. In the analysis, we focused on idea generation, stakeholder involvement, and the incorporation of stakeholder input within the development process. Overall, the generative techniques that were employed supported patients and HCPs with sharing their perspectives on pain treatment and relapse, which was in line with our hypothesis. Moreover, the techniques steered the conversations beyond stakeholders' primary responses, often resulting in a detailed account of their personal experiences with the treatment program and of their attempts to integrate treatment insights into their personal environment. In addition, system maps, personas, and prototypes enabled nonexperts to actively participate in design activities. A possible explanation for the successful engagement of stakeholders during the project is that experienced co-designers constantly translated hypotheses and abstract ideas into provotypes or prototypes. This method is particularly useful to provoke user reactions or to rapidly visualize an idea, which evokes interactions with an actual object rather than reflections on past experiences of hypothetical situations [39]. In addition, the used co-design materials helped to transform each location where co-design activities took place (eg, treatment facility or patient home) into a workshop environment that stimulated active participating and emphasized equality between all participants. This is especially important for health care settings, where conventional power relationships between patients and HCPs threaten effective cooperation during design activities [18,19].

With respect to stakeholder involvement, many different patients, HCPs, researchers, students, and designers participated during the study, which was also in line with our hypothesis. The stakeholder interactions mostly consisted of independent design activities that required low commitment and little effort. In contrast, the members of the core team remained active throughout the project, which increasingly created an imbalance in knowledge and involvement between the core team and other participants in co-design activities. This may explain why the role of the stakeholders gradually shifted from “user as partner”—where all participants within the sessions contributed as equals in the design activities—towards “users as subject”—where participants mainly provided expert opinions or performed delimited tasks (eg, usability testing) [49]. Consequently, the concepts underlying the intervention have been thoroughly grounded in stakeholder input and expertise, but the applicability of the current workbook operationalization within the treatment programs requires further testing to examine whether the current strategies fit patient preferences and can be integrated in treatment programs in the form of the current prototype.

This project shows similarities to the experience-based co-design (EBCD) approach, which aims to improve health care services by actively involving stakeholders to collect knowledge and experiences, to set priorities, and to develop solutions [18,50]. Although this project did not follow the 6 stages of EBCD, the overall objectives as well as the systematic partnership with patients, HCPs, designers, and researchers are alike. A notable difference was the focus within this project on actual prototype development throughout all phases, which promoted a solution-focused orientation for the participants. Alternatively, in EBCD, more emphasis is placed on ensuring that the collected patient experiences are received and understood by other stakeholders (eg, by showing a film of patient interview segments that reflect key themes), before continuing to developing improvements [18]. These differences illustrate the versatility of co-design and its potential to adapt to different design environments.

### Strengths and Limitations

The extensive documentation of the co-design activities allowed for a detailed reconstruction of the development process. Furthermore, during co-creation sessions, steering committee meetings, and the construction of the retrospective journey, representatives from all research groups were present, which resulted in a continuous integration of various perspectives during the project. However, we did not film or record any of the co-creation sessions. Although analyzing audio or video would have been time consuming, it would have provided further possibilities to observe stakeholder discussions during design activities and to include additional insights that we did not record.

During the project, we experienced a tradeoff between validating the outcomes of co-design activities and analyzing the results for the next iteration. For example, an additional co-creation session during the “Define” phase with different stakeholders could have cross-validated the outcomes of the initial session. However, given limited resources, this would have resulted in fewer development iterations in the remaining period. A key argument in favor of more iterations is that quickly integrating stakeholder input into subsequent sessions directly visualizes the value of their input [51]. However, a tendency towards more iterations increases the uncertainty to what extent the outcomes of this project can be generalized to the population [33].

### Future Recommendations

This study adds to the increasing number of initiatives that use co-design to structurally integrate contextual factors into the development of health care interventions (eg, [21,22,52,53]), which help bridge the gap from development to actual implementation [12,13]. When using co-design, it is important to relate the findings of the process to existing theories and treatments, for instance by using the behavior change technique taxonomy [23,47]. This strengthens the co-design approach by combining stakeholder evaluations with existing theory. Importantly, further integration between co-design and theory-driven approaches only becomes possible when using rigorous testing to evaluate the outcomes of the co-design process [13]. Consequently, an important next step in answering the question about whether co-design helps inform the development of health interventions will involve more examples of development projects. In these examples, co-design–based interventions are ideally subjected to experimental testing. Furthermore, we believe that future co-design projects in the health care domain should include detailed planning of activities and take lengthy medical ethical approval procedures into account [51].

### Conclusions

To acquire a better understanding of how co-design may benefit the development of interventions in the health care domain, examples of good practice are necessary**.** In this article, we presented such an example. By critically reviewing the value and function of a co-design project with respect to idea generation, stakeholder involvement, and incorporation of stakeholder input into the development process, we demonstrated how co-design contributed to the transition from ideas, via concepts, to prototypes.

## Appendix

Multimedia Appendix 1 Overview of the co-design development process.

## Abbreviations

EBCD: experience-based co-design
HCP: health care provider
PAR: participatory action research
