# Supplementary material for: Innovation in Pain Rehabilitation Using Co-Design Methods During the Development of a Relapse Prevention Intervention: Case Study
Source: J Med Internet Res. 2021 Jan 20;23(1):e18462. doi: 10.2196/18462 (PMC7857944; doi:10.2196/18462)
Supplement: Multimedia Appendix 1 [file jmir_v23i1e18462_app1.pdf]

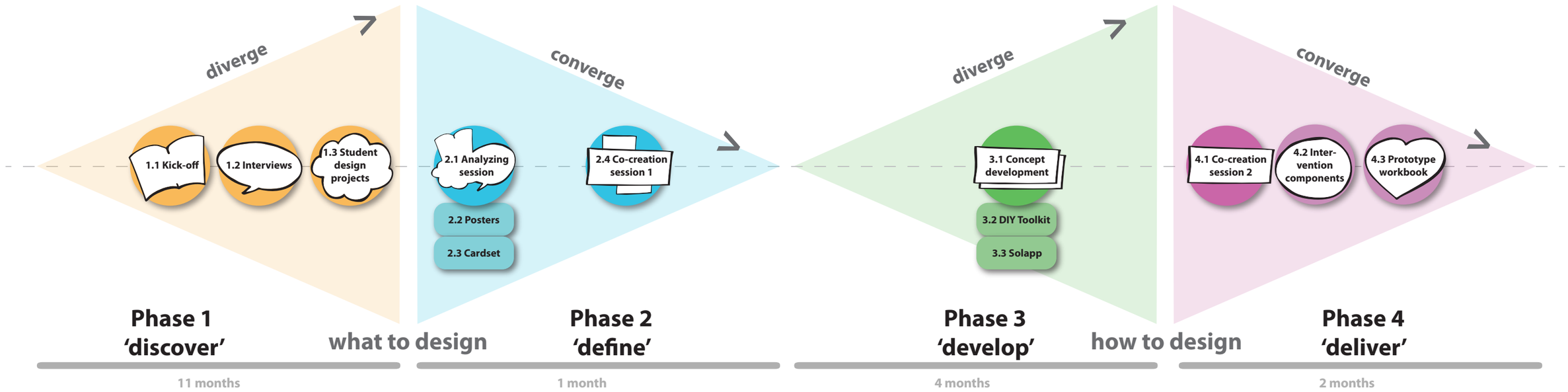
diverge
converge
diverge
converge
1.1 Kick-off
1.2 Interviews
1.3 Student design projects
2.1 Analyzing session
2.2 Posters
2.3 Cardset
2.4 Co-creation session 1
3.1 Concept development
3.2 DIY Toolkit
3.3 Solapp
4.1 Co-creation session 2
4.2 Inter-vention components
4.3 Prototype workbook
Phase 1 'discover'
what to design
Phase 2 'define'
Phase 3 'develop'
how to design
Phase 4 'deliver'
11 months
1 month
4 months
2 months

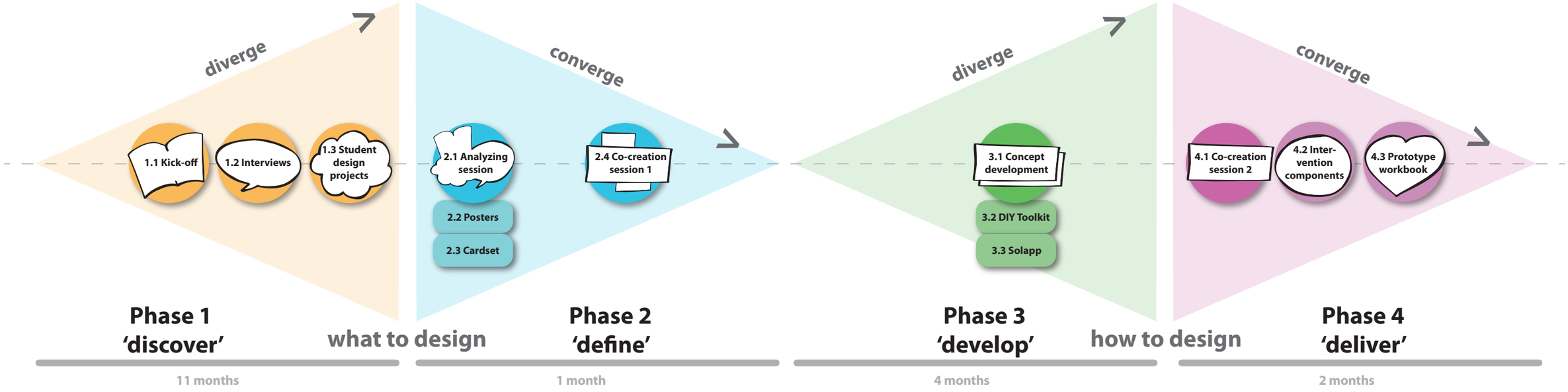

Discover involves an exploration of the problem by spending time with stakeholders and learning to understand their perspectives and experiences.

Define relates to the integration of the acquired insights into various separate concepts that each provides a specific direction for potential solutions.

Develop is concerned with exploring various potential strategies together with stakeholders that be a solution to the problem.

Deliver converges the potential strategies towards an end result. In our case: a paper prototype that contained both strategies that were explored in the develop phase.

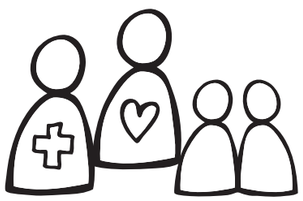

SOLACE

SELF MANAGEMENT IN CHRONIC PAIN STRATEGIES

Phase 1 ‘discover’

1.1 Kick off

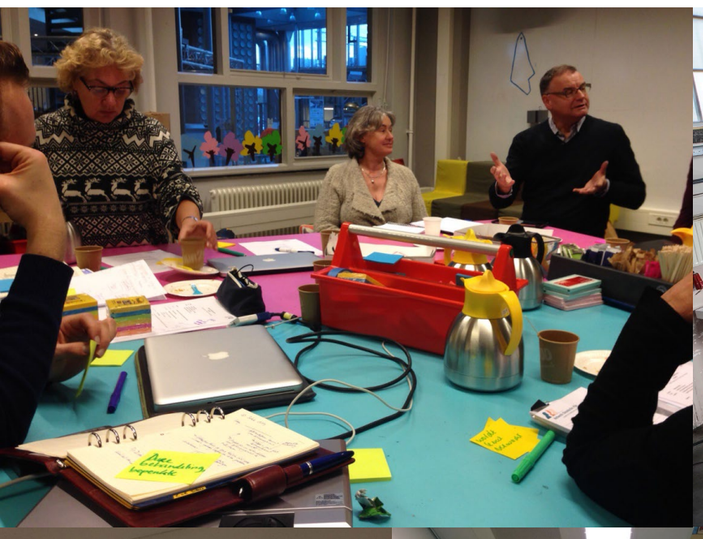

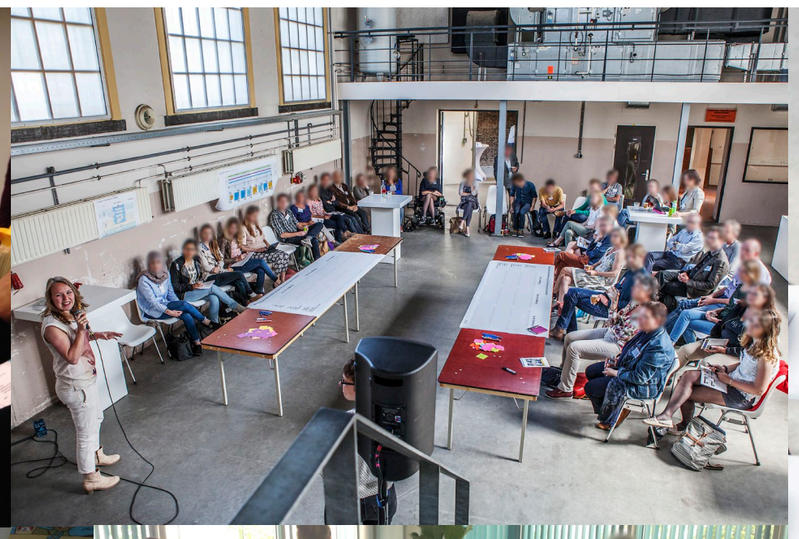

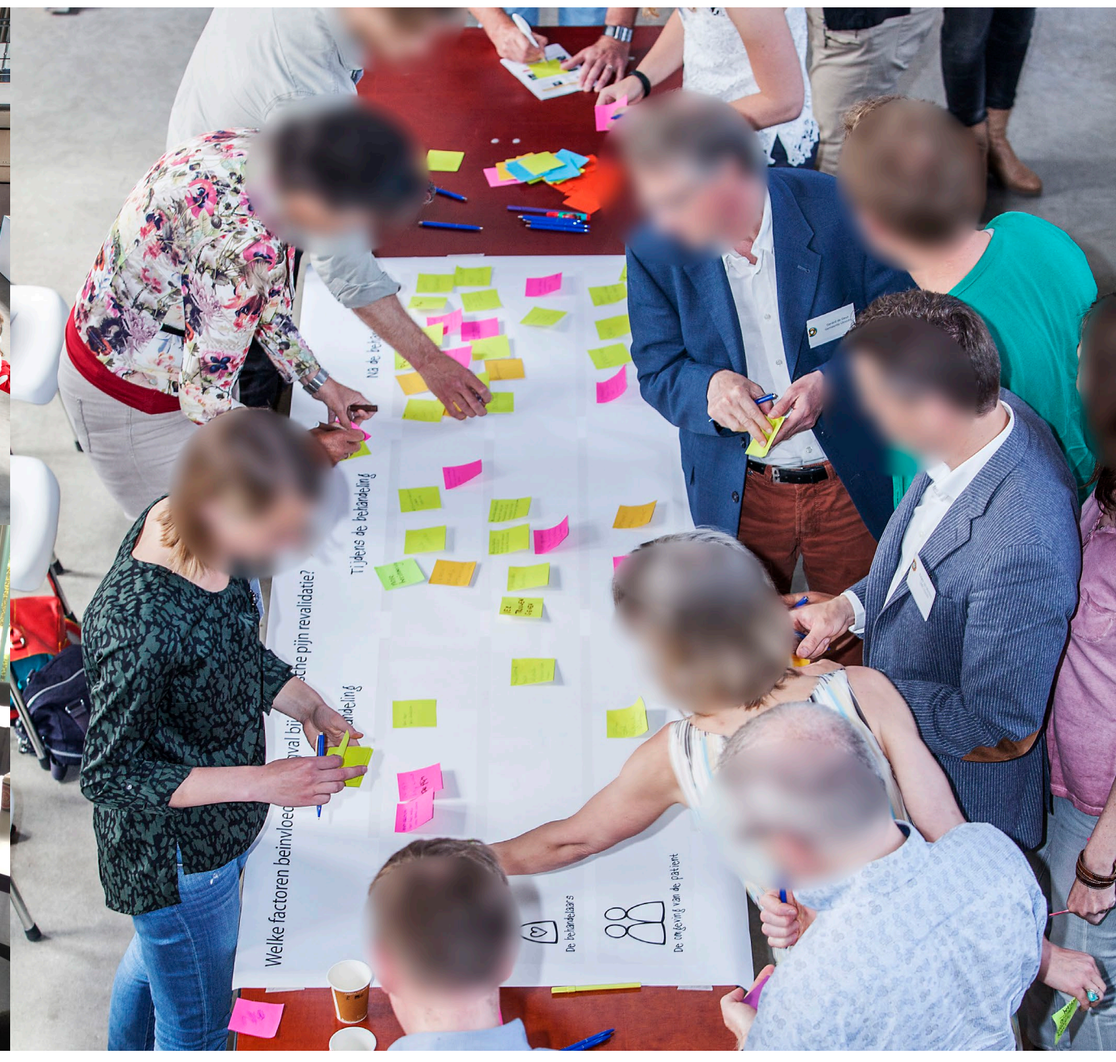

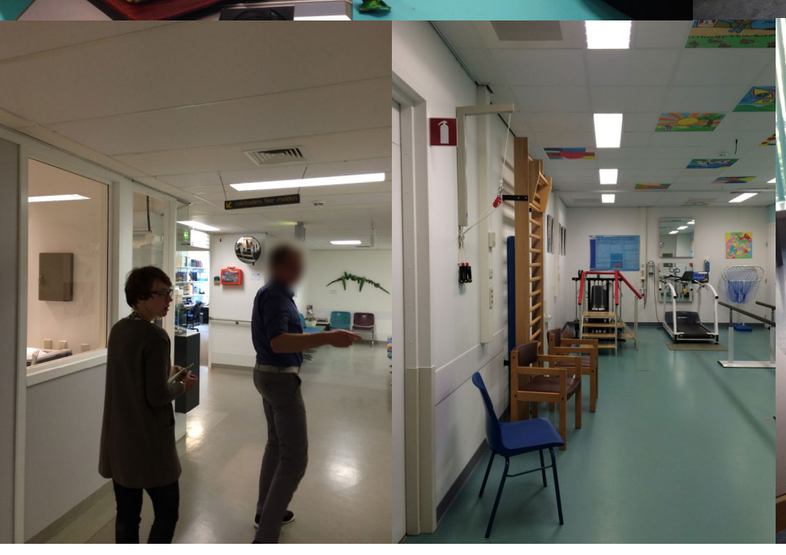

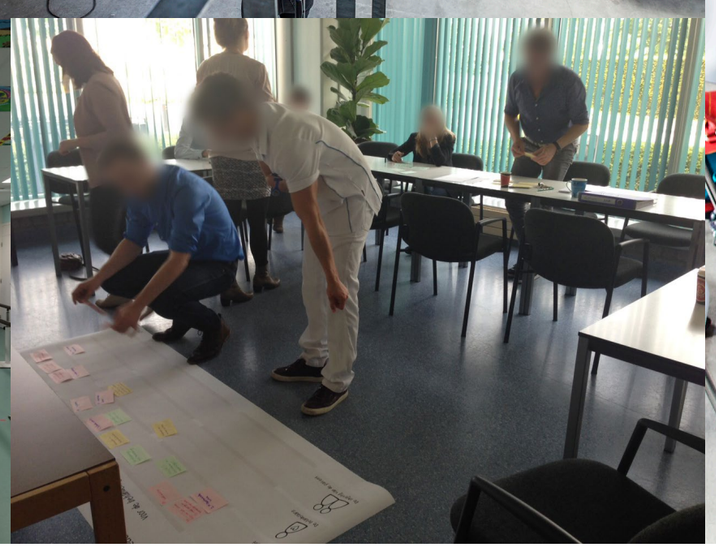

An illustration of the kick-off sessions, including location visits, the first consortium meeting, and the formulation of stakeholders’ initial ideas on relapse. For this purpose, we used a patient journey, that not only facilitated the generation of insights, but also transformed the room into a design space.

Empty Journey map

Example of a generative technique that facilitated participants to share their initial ideas and experiences that were related to relapse. Time (before, during and after treatment) is depicted on the x-axis, and important stakeholders (i.e. patient, healthcare providers and their immediate social system) are depicted on the y-axis.

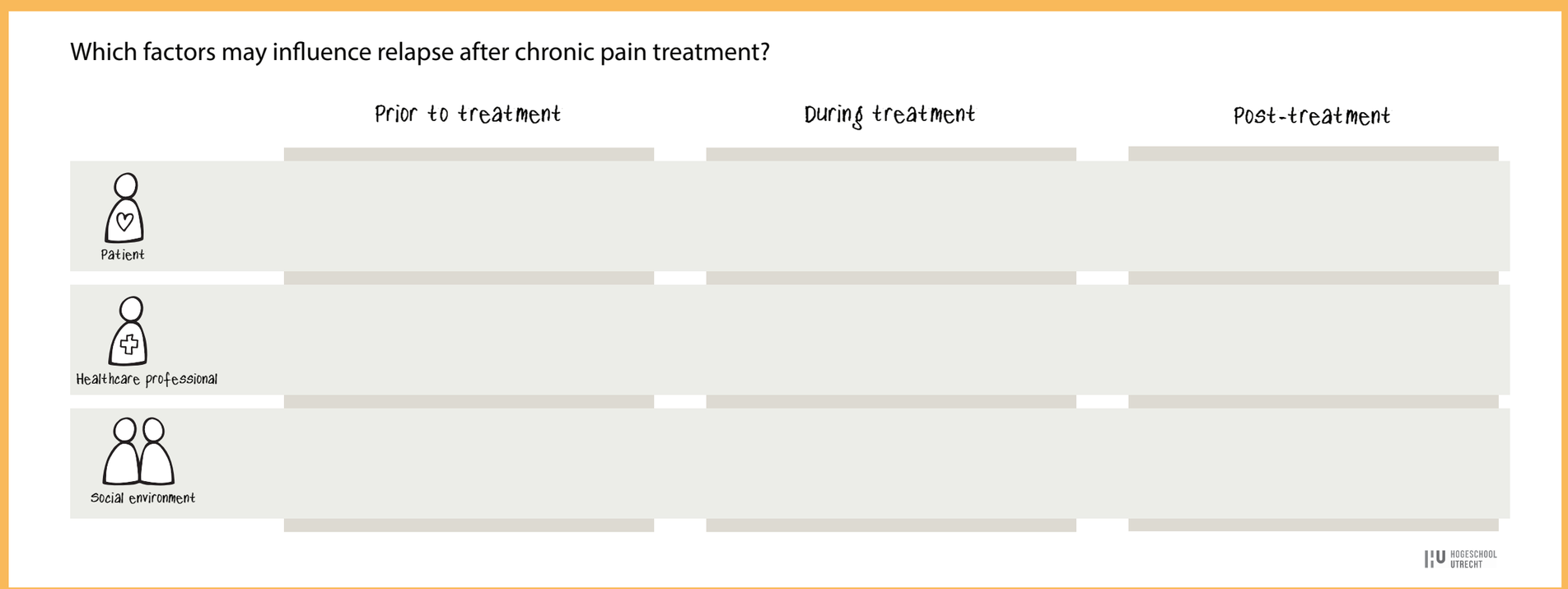

SOLACE

SELF MANAGEMENT IN CHRONIC PAIN STRATEGIES

Phase 1 ‘discover’

1.2 Interviews

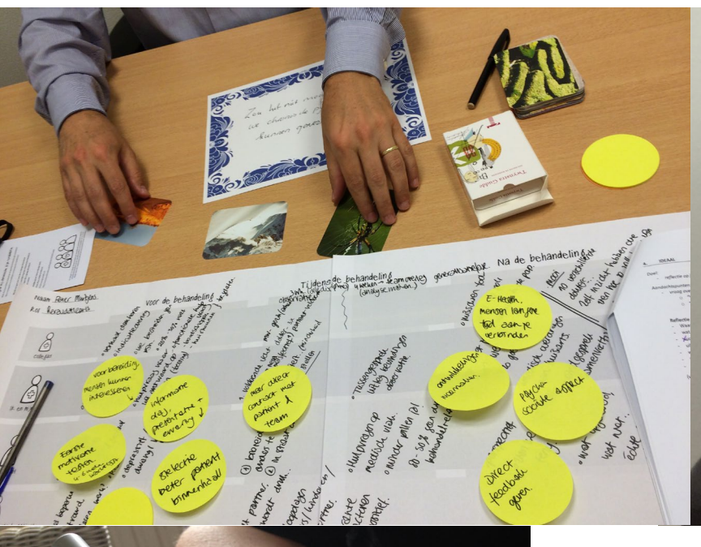

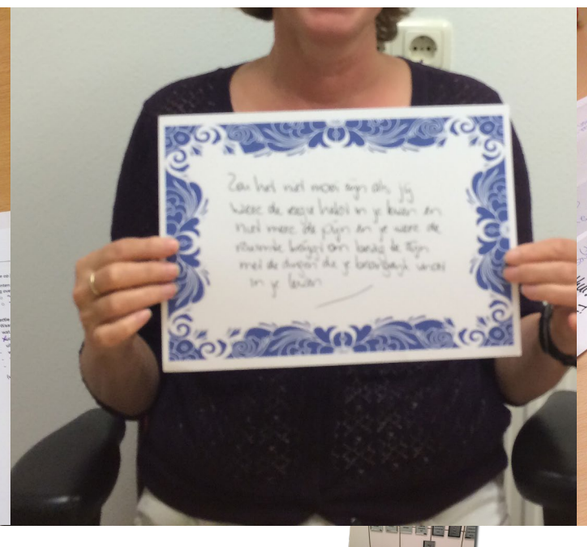

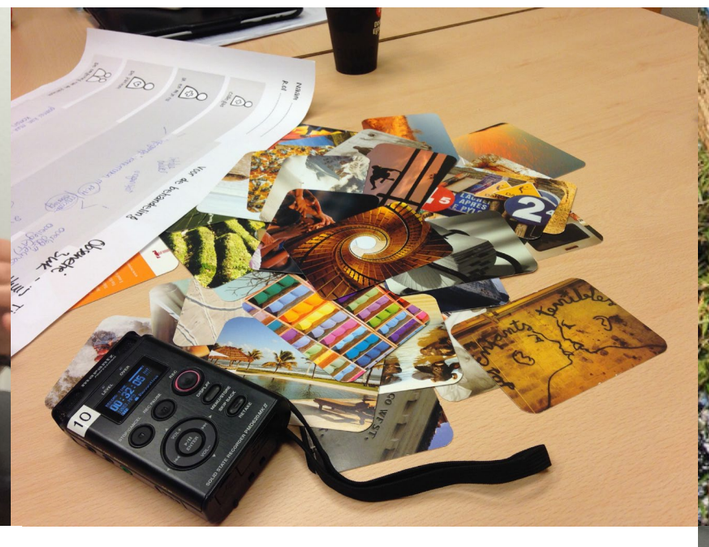

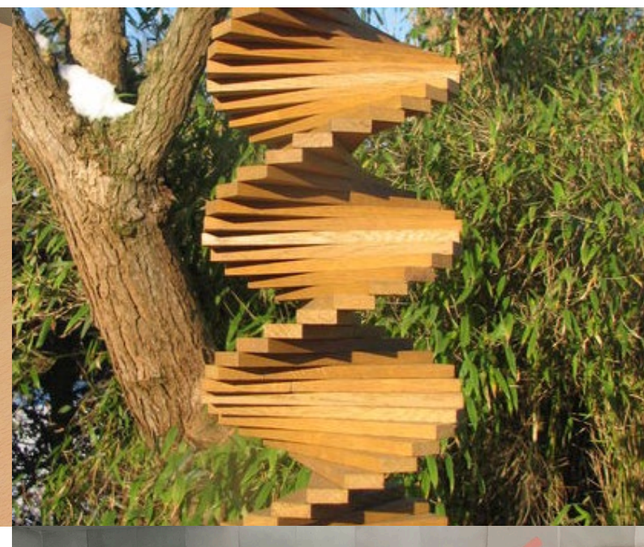

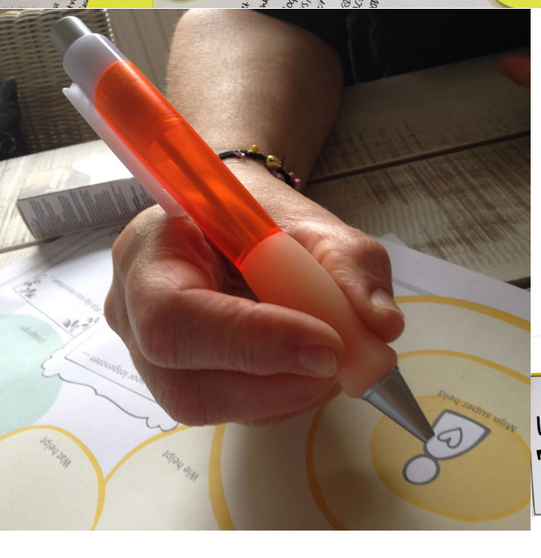

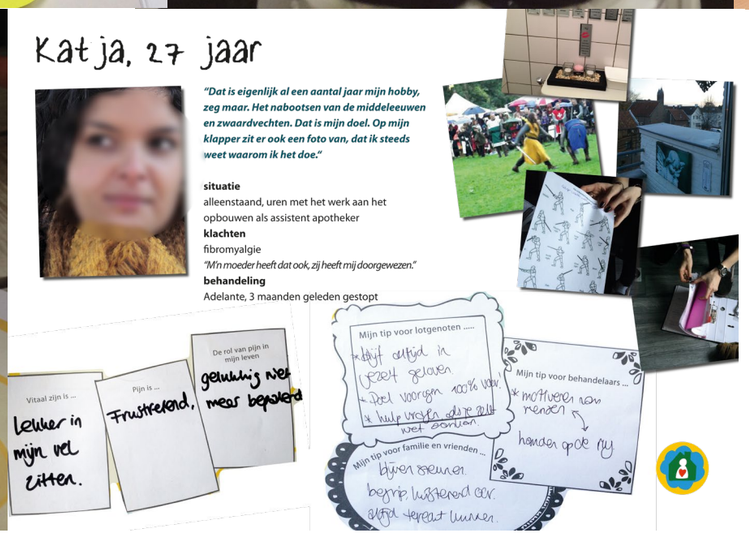

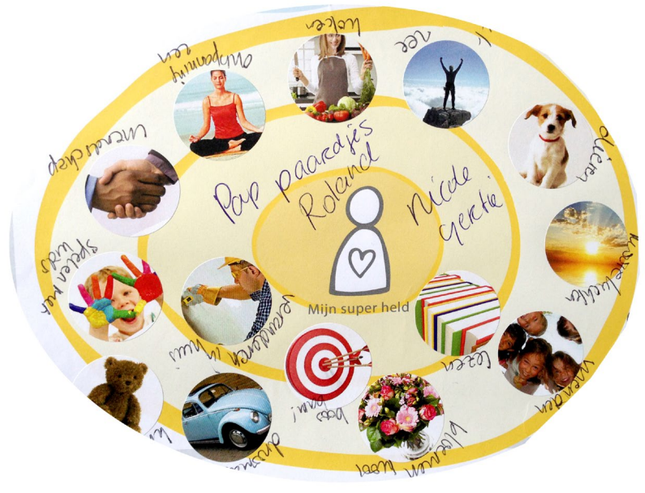

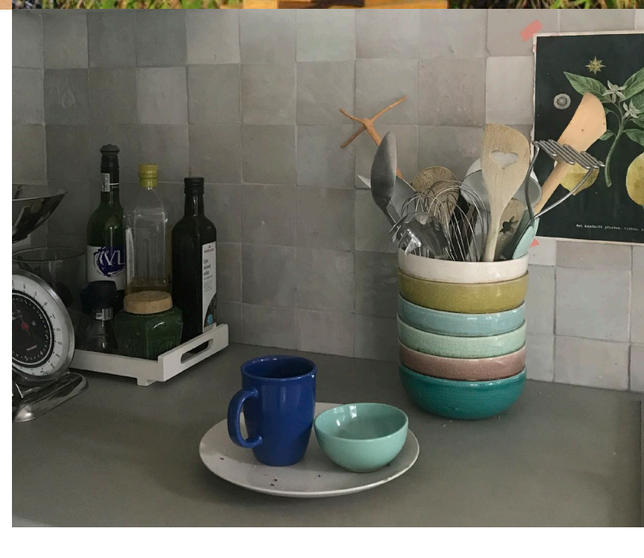

Illustration of stakeholder interviews, including the ‘ambiguous images’ that were used during the introduction, journey maps and a an assignment to write down a personal desire (“wouldn’t it be nice if …”) related to improving the care for patients with chronic pain. Patient were interviewed in their personal context.

Generative techniques for patient interviews

Prior to the interview, a patient received a ‘sensitizer’ (left) to pre-activate thoughts and ideas related to their experience (e.g. description of a day were they felt particularly well or restricted by the pain). During the interview both the patient and interviewer collaborated to construct a patient journey map that laid on the table (right). The stickers aimed to elicit associations or to bring up tacit knowledge.

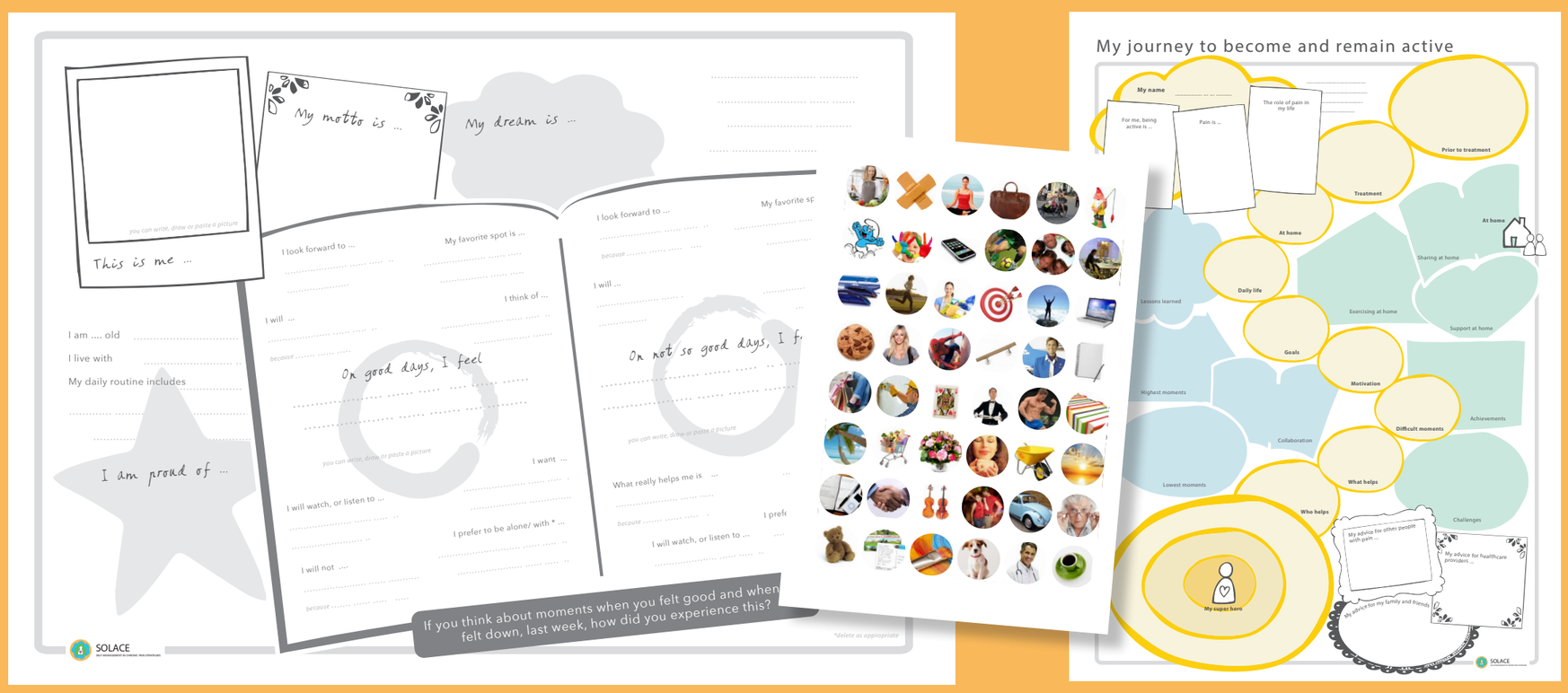

SOLACE

SELF MANAGEMENT IN CHRONIC PAIN STRATEGIES

Phase 1 ‘discover’

1.3 Student design projects

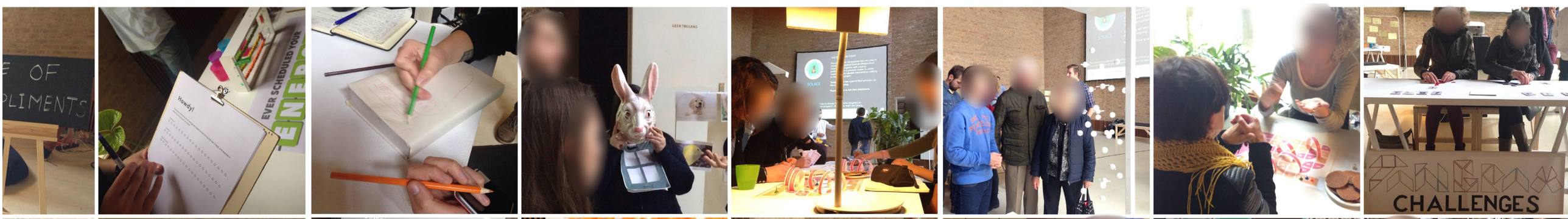

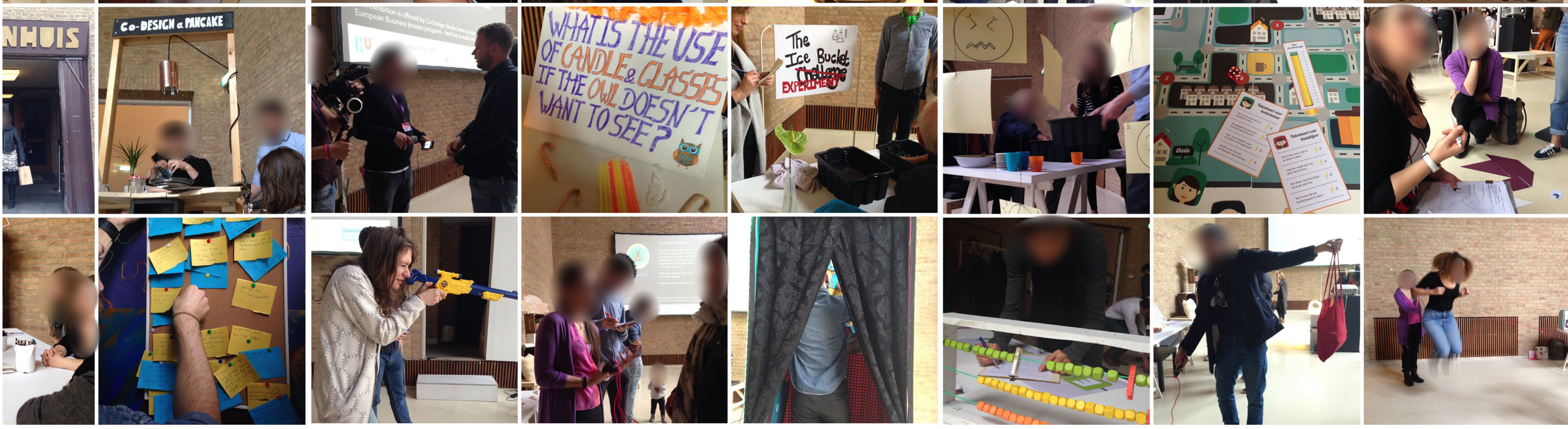

Example of various student teams that tested specific hypotheses related to relapse. Participants (e.g. bystanders or patients) interacted with provotypes to acquire insights.

Provotypes

Two examples of provotypes that were developed and used by the student teams. ‘Spirit’ was a board game were patients and their families had to cooperate to successfully complete a normal day, including work, school and all required tasks. Importantly, participants had to switch roles and play a different family member. This tested the idea that sharing details of coping with pain in daily life with family would reinforce empathy and mutual understanding. In ‘Co-design a pancake’, patients and significant others had to collaborate to bake a pancake, which was further stimulated by modifying the kitchenware (e.g. a ladle with two handles). This tested the assumption that pain could interfere with equality as partners.

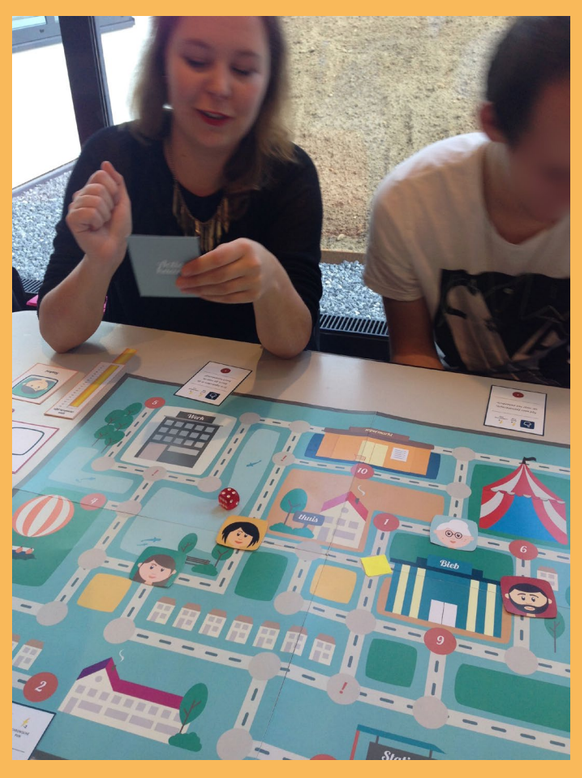

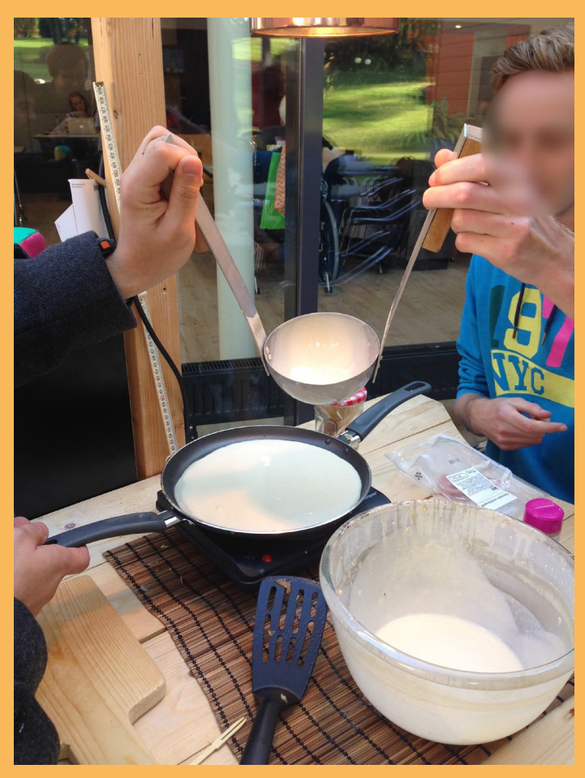

Phase 2 'define'

2.1 Analyzing session

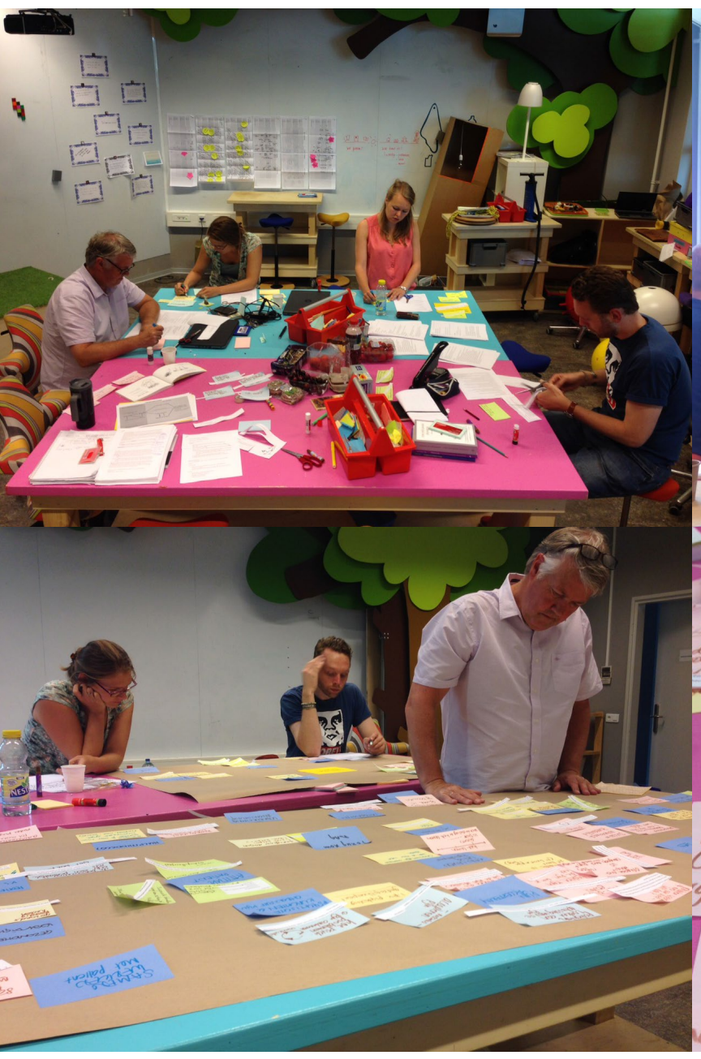

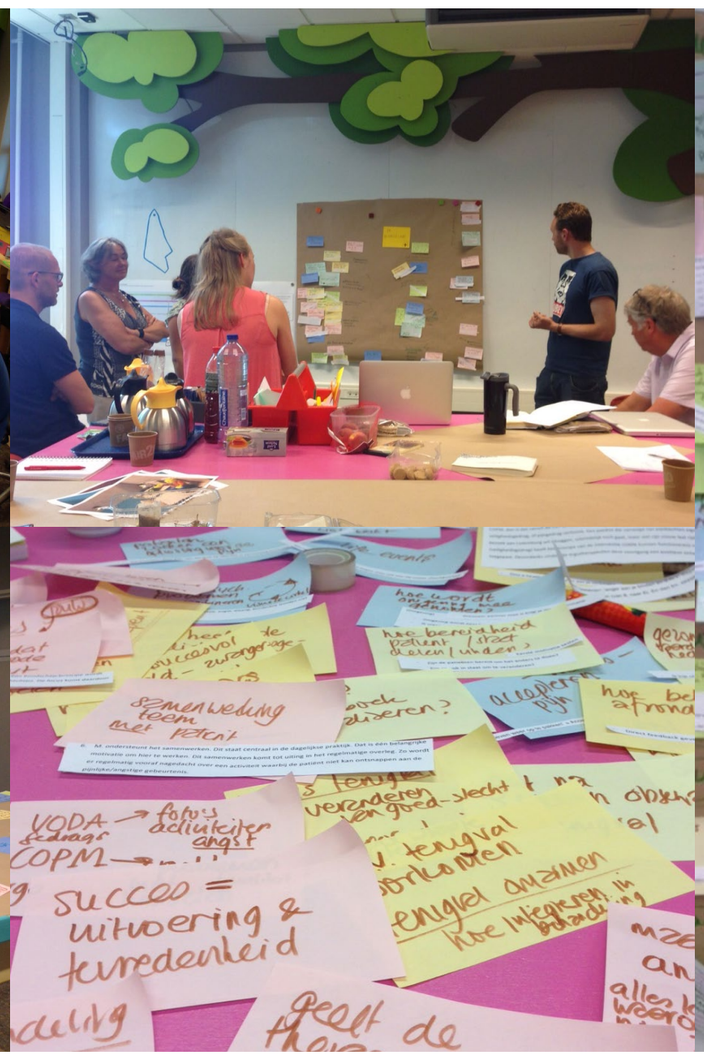

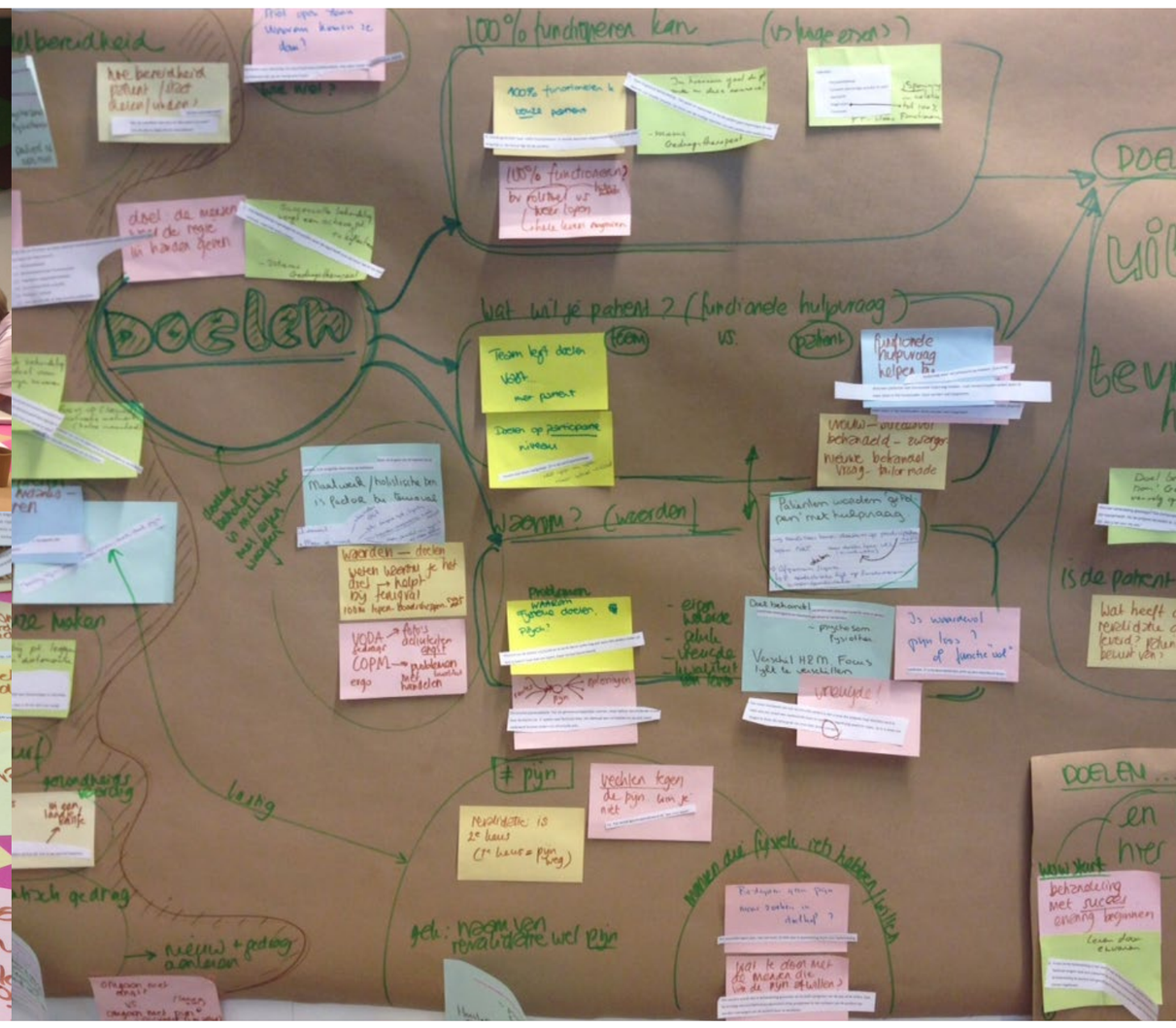

Overview of analysing sessions with the core teams to organize and cluster the interview and kick-off data. Relevant data segments were placed on post-its and organized around emerging insights and clustered in overarching themes, in shared within the team.

System map

The system map for the first co-creation session consisted of 8 posters that each contained a theme (rephrased as a question), subthemes and corresponding quotes and pictures. In addition, 74 stimulus cards were developed that each contained a specific insight or principle.

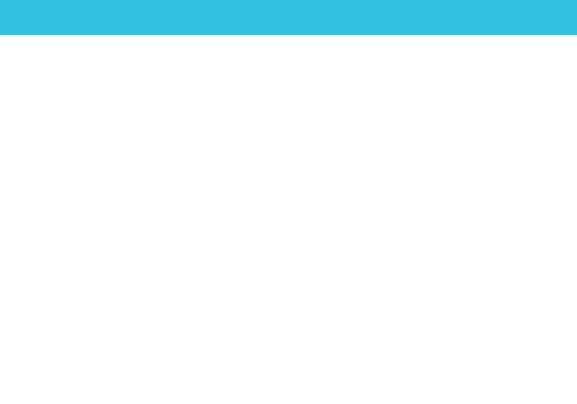

Expressing frustration is important.

During the Dutch Design Week, a women told us that she learned to cope with her pain by writing poems.

Team Up

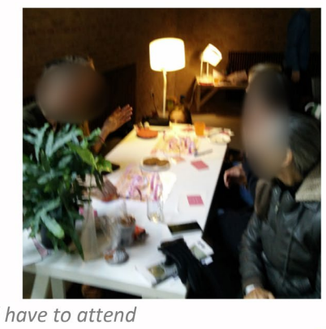

"sometimes, you will have to attend to the pain, to be able to enjoy yourself later."

Anonymus

Co-design minor

SOLACE

SELF MANAGEMENT IN CHRONIC PAIN STRATEGIES

Phase 2 'define'

2.2 Posters

Overview of themes, subthemes and exemplary quotes of the data that was collected in the 'discover' phase.

Themes	Subthemes	Quotes
**1. Emptiness after treatment**<br>*How will you cope with being on your own, after treatment? How do you keep believing in yourself and remain focused on important treatment insights?*	**1. Emptiness**<br>**2. Pride**<br>**3. Responsibility**<br>**4. Effective relationship with HCPs**<br>**5. Confidence**<br>**6. How to remain motivated to remain focused on treatment insights by yourself**<br>**7. Post-treatment contact between patient and HCP**	*"I am proud of my children and grandchildren. And I am also proud of what I achieved during the treatment program."*<br>*– patient, 1.2*<br><br>*"Confidence is an important topic during treatment. It helps if you show that you are confident that a patient is able to achieve his or her goals."*<br>*– HCP, 1.5*
**2. Goals and values**<br>*How do you maintain focus on your goals and values? How do you remain engaged? How do you stay on track rather than being distracted by the pain?*	**1. Remembering goals and values**<br>**2. Differences between treatment centre and personal environment**<br>**3. Structured daily schedule**<br>**4. Planning daily activities**<br>**5. Motivation**<br>**6. Continuing pain interference**	*"Walking was my most important goal. Why? Because it gives me freedom."*<br>*- patient, 2.1*<br><br>*"You will not win the battle against the pain.. wouldn't it be nice if to focus shifted from pain to general enjoyment of life?"*<br>*– HCP, 2.6*
**3. Coping with a setback**<br>*What if things are not going well? Or if you forgot how to manage certain situations? What if you experience a setback?*	**1. What if things are not going well**<br>**2. Live events and new injuries**<br>**3. Remembering treatment insights**<br>**4. Getting up after a setback**<br>**5. Asking for help**<br>**6. Talking with others**<br>**7. Integrating the topic of relapse in the treatment**	*"She [therapist] taught me to use the [graded activity] schedule for a couple of days, in case that I fall back into old habits."*<br>*- patient, 3.4*<br><br>*"I think it is a good thing if patients experience a setback during treatment. Than you can use this experience to make prevention plans in anticipation of future events."*<br>*- HCP, 3.7*
**4. Skills to prevent relapse**<br>*What are important skills to prevent relapse? How can you train or promote these skills? How do they become a part of you?*	**1. Creativity**<br>**2. Impulsivity and courage**<br>**3. Positivity, joy and humor**<br>**4. Resilience, acceptance, letting-go**<br>**5. Reflecting**<br>**6. Self-compabssion**<br>**7. Distraction, shifting focus**	*Creativity is an important trait. If a strategy does not work, patients will have to find new solutions*<br>*- HCP, 4.1*<br><br>*"I learned to have self-compassion. Before treatment, I used to punish myself, but now, I don't do that anymore. If you like yourself, you are also prepared to take good care for yourself."*<br>*- patient, 4.6*
**5. Involving significant others**<br>*How do you explain your treatment experiences to your significant others? How do you communicate that you have changed and what your new goals are?*	**1. Sharing experiences**<br>**2. Support and understanding**<br>**3. Barriers within the family**<br>**4. Habits**<br>**5. Attention**	*"A patient can change, but if the social environment is not sufficiently supportive the chance of relapse will increase. That's why it is important to involve the family during treatment."*<br>*- HCP 5.2*<br><br>*"My husband always wanted to care for me and to ensure that nothing bad happened. That was difficult for me, I had to ask him to stop interfering."*<br>*- patient, 5.3*
**6. Personal boundaries, pacing and monitoring energy**<br>*How do you remain true to yourself and communicate your boundaries? How do you manage your energy levels and remain relaxed?*	**1. Rest**<br>**2. Making decisions and setting personal boundaries**<br>**3. High demands**<br>**4. Saying 'no'**<br>**5. Energy management**<br>**6. Limited by pain**	*"The treatment is successful if a patient regained control over his life. Not the pain is central, but his aims and desires in life."*<br>*- HCP, 6.4*<br><br>*"Planning my daily schedule in advance was useful for me. I learned how to distribute my time and energy by adhering to the schedule."*<br>*- Patient, 6.5*
**7. Improving after treatment**<br>*How can you continue to improve after treatment?*	**1. End of treatment**<br>**2. Treatment successes?**<br>**3. Setting new goals**<br>**4. Back to the old personal environment**	*"We used to celebrate the end of a treatment period with a party and a couple of beers. But we don't do that anymore. We do have a conversation about what we learned and how they experienced the program."*<br>*– group coach, 7.1*<br><br>*"After fifteen years of pain, I immediately wanted to check if I was ok, after treatment. I started kickboxing, which actually went quite well."*<br>*- patient, 7.3*
**8. Insight into own behaviours**<br>*How do you acquire insight into your own behaviour and how things are going? How do you monitor your current level and your progress? How do you organize feedback and how should you ideally respond to this?*	**1. Self-insight**<br>**2. Monitoring behaviour**<br>**3. Receiving feedback**	*"I learned to have more confidence in my body. That you actually can do whatever you want, but sometimes you need to do it slightly different than the rest."*<br>*– patient, 8.2*<br><br>*"Innovations and eHealth are opportunities to monitor patients after treatment and to signal relapse. That could be combined with online sessions with a psychologist."*<br>*- HCP, 8.2*

Example of a poster that was created for each of the themes (headings translated).

WHAT IF THINGS ARE NOT GOING WELL

Er komt een moment dat het niet meer goed gaat, want doen mensen dan? Vragen ze om hulp? Bellen ze de behandelaar? Kijken ze terug in het geleerde? Gaan ze nieuwe hulp zoeken? Hoe hen hierbij te helpen?

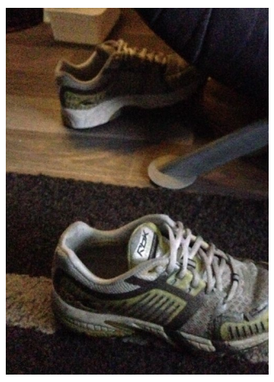

"Patiënten worden sterk geadviseerd om te bellen of te mailen als er iets misgaat. Hier wordt echter zelden gebruik van gemaakt."
- ergo/ gedragstherapeut/ onderzoeker

"Wanneer patiënt weer in circuit komt, bezig zijn met pijn. Wanneer ze bijvoorbeeld weer naar middelen grijpen. (Medicatie, braces, rolstoelen) ." - psycholoog

"Risico's van terugval is dus dat er generalisatie plaatsvindt. Niet meer de meewaarde inzien."
- Gedragstherapeut

"Ik begon me eigenlijk wel een beetje terug te trekken. Bij mij thuis zagen ze het al, hé mam zegt weer liever niks."
- vrouw (55)

"Momenten dat het erger wordt, is als ik stress heb. Gister had ik echt zo'n hoofd van de spanning."
- vrouw (41)

"Kijk ik weet wat ik mag doen en niet mag doen en eigenlijk hou ik me daar gewoon aan. Ik heb wel nog bultjes en kuiltjes maar het zijn geen bergen en dalen meer ik zit eigenlijk redelijk recht op één lijn. Dat is wel anders geweest."
- vrouw (53)

LIVE EVENTS AND NEW INJURIES

Grote dingen veranderen in het leven, zoals bijvoorbeeld het krijgen van een kind, hoe in deze nieuwe situaties weer goed te functioneren? Wat als er nieuw letsel ontstaat, een nieuwe situatie, waar de behandeling niet op in heeft gespeeld. Zoals een auto ongeluk of een sport blessure. Wat nu te doen?

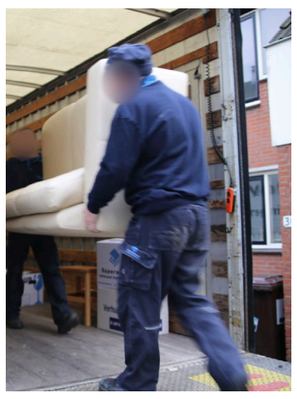

"Bij terugval gebeurt er iets in het leven van de patiënt, waardoor hij tijdelijk afwijkt van zijn behandelprincipes en waarbij hij daarna niet meer in staat is om deze op te pakken."
- Ergotherapeut

"In sommige gevallen komt iemand terug met een concrete hulpvraag (bijv. net een kind gekregen, en zorg voor het kind optimaliseren na succesvolle revalidatie)."
- Ergotherapeut

"Valkuilen zijn als er lichamelijk iets extras bijkomt gebroken been b.v., of ziek worden." - Gedragstherapeut

"Soms ben ik echt heel verdrietig, dan ga ik meestal in het veld lopen en dan doe ik eens flink schelden. Dan zeg ik van 'godverdomme' en dan op een gegeven moment heb ik dan zo iets van 'zo, nou is het klaar' en dan ga ik weer verder."
- vrouw (41)

"Het is voor mijn natuurlijk wel een aandachtspunt die geblesseerde knie. Mijn knie moet niet de nieuwe rug worden. Maar bij mijn rug was niks aantoonbaar kapot, en bij mijn knie is nu natuurlijk wel gewoon aantoonbaar kraakbeen beschadigd. Ik moet nu wel uitkijken dat ik daar niet weer heel erg over na ga denken. En doktoren behandelen het ook anti-Maastricht, 'U mag niet sporten' en dan moet ik wel uitkijken, echt een valkuil zeg maar."
- man (40)

WHAT IF THINGS ARE NOT GOING WELL? OR IF YOU FORGOT HOW TO MANAGE CERTAIN SITUATIONS? WHAT IF YOU EXPERIENCE A SETBACK?

REMEMBERING TREATMENT INSIGHTS

Bij de behandeling heb je veel geleerd, maar in het dagelijks leven lijkt dat soms weer ver weg. Hoe houd je het geleerde vast? Hoe herinner je je weer hoe goed je je voelde? Hoe goed je het deed? Wat waren WOW momenten? Hoe lees of voel je het terug? Een brief? Een video? Een boek? Ansichtkaarten? Gelukskoekjes?

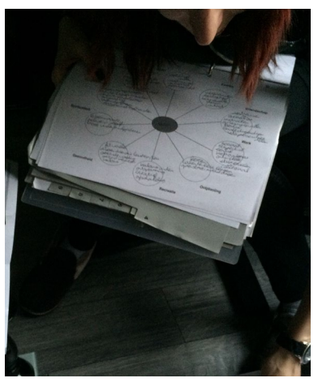

"Wij hebben ook wel eens een kutdag, dat is naar mijn idee geen terugval. Terugval is dat wat je hebt opgebouwd niet meer lukt."
- groepsbegeleiding

"In een eerste behandeling is vaak van belang om een "wow ervaring" te creëren. Patiënten krijgen vaak een toename van de klachten na afloop van de eerste behandelsessies en als de behandeling de patiënt het gevoel geeft dat er direct een grote stap is gezet, dan worden die minnen opgeheven." - ergo/gedragstherapeut/onderzoeker

geprint, en toen kreeg ik een spontaan idee, ik ga hem gewoon op mijn klapper plakken. Zodat elke keer als ik mijn klapper zie, ik denk 'Oh ja, daar ben ik voor, daar werk ik voor.'"
- vrouw (27)

"Als ik in mijn klapper keek, dan dacht ik 'Wow, ik ben toch wel ver gekomen!'. Dan zag ik bijvoorbeeld bij het opdrukken staan '5 minuten'. Dat ik er echt van op keek en dacht van 'zo', dat had ik niet verwacht."
- vrouw (27)

GETTING UP AFTER A SETBACK

Als het niet goed gaat, hoe komt je daar dan weer uit? Hoe ben je je bewust van hoe het gaat? En hoe herpak je je weer? Gebruik je weer het gene wat je geleerd hebt?

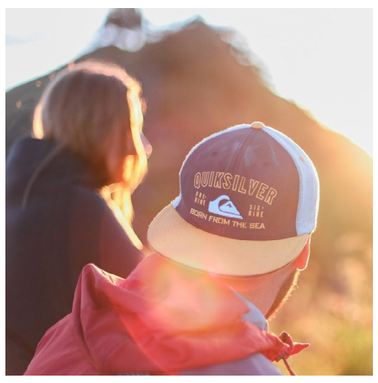

"Tijdens de laatste sessie lopen we de score van de doelen na, leggen we de foto's weer neer." Bespreken het terugval preventieplan. De patiënt vult zelf actief het plan in. Wat ze doen is situaties die terugval kunnen veroorzaken daar bespreken en ze bespreken vooraf hoe ze dan anders kunnen denken.
- Gedragstherapeut

weer wegglijd in je oude gedrag, moet je even een paar dagen zo'n tijdschema bijhouden. Dan kan je zien waar ga ik de fout in, wat kost me te veel energie en wat kan ik daar mee doen, en zo moet je dan eigenlijk alles gaan benaderen."
- vrouw (29)

"Dan wordt het zwaar, want je bent dan weer alleen en dan ga je weer. In het begin ga je dan weer over je grens heen, omdat je het dan toch gedaan wilt hebben. Dan loop je er juist tegen aan. Wat wel helpt is gewoon veel met mijn vriend en mijn dochter praten."
- vrouw (48)

ASKING FOR HELP

Hoe weet je wanneer je hulp moet vragen? Wat is de ondergrens? En waar? Sommigen spreken over groene en rode pijn. De patient moet weten wanneer en waar hulp te vragen. Maar wat als dokters andere dingen zeggen dan het geleerde bij de revalidatie? Hoe hier op te reageren?

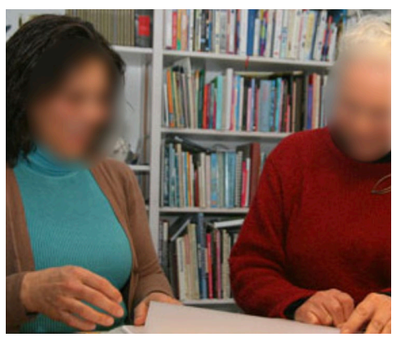

"Concreet: minder naar school, minder sociale contacten en minder sporten. "Als het niet belangrijk is, dan is het geen terugval."
- Gedragstherapeut

"Uitspraak artsen over nieuwe ideeën voor behandeling, bv gen therapie. 'Heb je dat al eens geprobeerd?' Hoe moet de patient hier op reageren?"

"De tuin werd mij te zwaar, maar ik wilde het zo graag doen. Uiteindelijk heb ik iemand anders geregeld die het voor me kon doen. Maar ja, dan kom je op het punt, je moet hulp vragen. Iets wat bij mensen met chronische pijn moeilijk gaat." - vrouw (29)

"Blijf altijd in jezelf geloven. Probeer een doel voor ogen te vinden en ga daar de volledige 100% voor. En wees niet bang om ook andere mensen om hulp te vragen, je kunt het soms zelf niet aan. Dat is denk ik wat ik zelf ook geleerd heb, dus dat zou ik ook aan andere meegeven." - vrouw (27)

"Ik probeer gewoon eerlijk te zijn als iemand me vraagt hoe het gaat. Vroeger was het zo, ja, het gaat wel goed, nu zeg ik het eerlijk." - vrouw (29)

"Maar ik kan me voorstellen dat er nog wel eens vragen komen die je misschien even snel kwijt wil en dat je dan iemand kan mailen of dat er online een vragenportaal of iets is." - vrouw (29)

TALKING WITH OTHERS

Je uitten, eerlijk zijn over je gevoel, dat helpt. Weten dat je iemand mag bellen als je dat nodig hebt. Toch is het lastig als je je niet goed voelt. Hoe kan je mensen hierbij helpen?

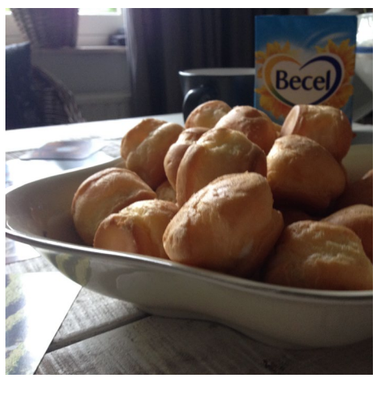

"Bij het terugkom gesprek geven patiënten in eerste instantie vaak aan dat ze nog steeds een heleboel pijnklachten hebben. Als je doorvraagt dan blijkt dat er op functioneren vaak wel winst is geboekt. Bij de terugkom dag worden deze verbeteringen vaak positief bekrachtigd."
– revalidatie arts

"Ik heb geleerd, zeg gewoon wat je op je lever heb, blijf daar niet mee rond lopen, want je heb daar jezelf mee. Dan loop jij vanavond weer met zo'n koppijn en morgen heb je weer pijn in je ledematen alleen doordat je dan de hele nacht weer loopt te denken. Gooi het eruit, dan ben jij er vanaf en die ander doet er maar mee wat die ermee wil doen."
- vrouw (48)

"Het heeft me geholpen om meer over me zelf te gaan vertellen en te laten zien."
- vrouw (27)

"Het was niet eens dat ik de pijn benoemde, want dan had ik zo iets van, dan hoort ze me weer zeiken daarover."
- vrouw (29)

INTEGRATING THE TOPIC OF RELAPSE IN THE TREATMENT

Wat is terugval? Een slechte dag? Meerdere slechte dagen? Als je als patient het geleerde niet meer gebruikt. Deze momenten zullen komen. Juist dan moet je ingrijpen. Hoe kan dit geintergreerd worden in de behandeling?

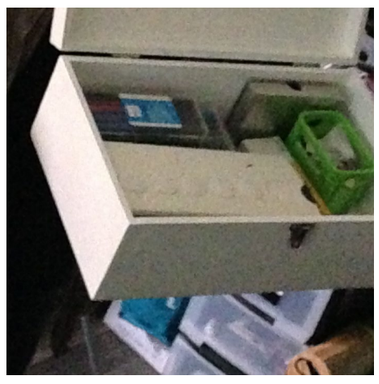

"Hopelijk valt iemand terug tijdens de revalidatie. Daar kan je dan mee aan de slag. Je kijkt op zo'n moment terug naar wat er is gebeurt. Je stelt patiënten gerust – "dit overkomt iedereen wel eens" – en je maakt een plan voor de toekomst."
- Psychosomatisch fysiotherapeut

dag en dan na drie maanden. En dat vind ik gewoon veel te weinig. Ik had graag nog één dag in de week gesproken met een maatschappelijk werker, een fysio of een ergo. Dat ze je kunnen helpen met wat je nog tegenkomt thuis."
- vrouw (48)

"Ik merk dat wel een hele grote valkuil is geweest na de behandeling. Er is niemand meer die je begeleidt of zegt van 'joh, je moet daar wel even op letten'. Je weet hoelang je behandeling duurt, je weet dit is mijn laatste week, je bent er eigenlijk zelf al mee bezig, maar toch wil je graag meer handvatten voor thuis en dit en dat."
- vrouw (48)

SOLACE
SELF MANAGEMENT IN CHRONIC PAIN STRATEGIES

Co-creatie sessie - februari 2016

Phase 2 'define'

2.3 Cardset

Examples of four stimulus cards that where developed for the first co-creation session.

Relapse: a struggle against old habits?

A habit is a type of behaviour (e.g. resting) that is executed automatically and effortlessly in respons to an associated stable situational cue (e.g. pain). The stronger this association, the stronger the impulse to perform the corresponding habitual behaviour.

New treatment goals

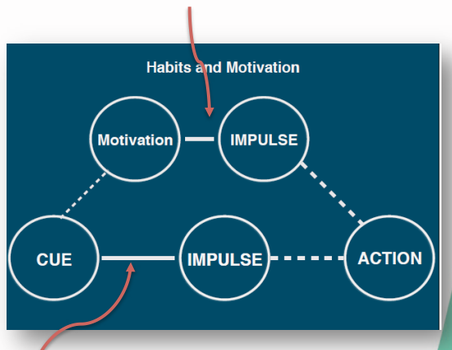

... Versus old habits.

Gardner, 2014

Literature - behaviour change

Title of your idea or insight

Attach an illustration, picture or quote that is associated with your idea or insight

describe your idea or insight here

Sender

Barriers to self-management

- Lack of support of family and friends
- Limited financial means
- Depression
- Ineffective pain management strategies
- Activity avoidance due to pain

Bair (2008)

Literature – chronic pain

Expressing frustration is important.

During the Dutch Design Week, a women told us that she learned to cope with her pain by writing poems.

Team Up

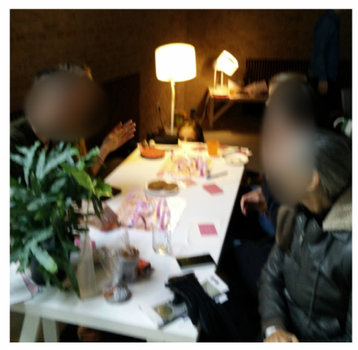

"sometimes, you will have to attend to the pain, to be able to enjoy yourself later."

Anonymus

Co-design minor

SOLACE

SELF MANAGEMENT IN CHRONIC PAIN STRATEGIES

Phase 2 'define'

2.4 Co-creation session 1

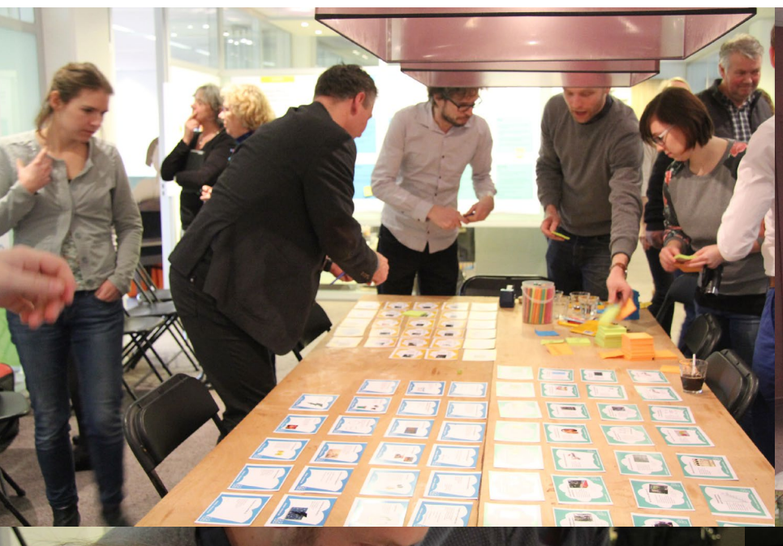
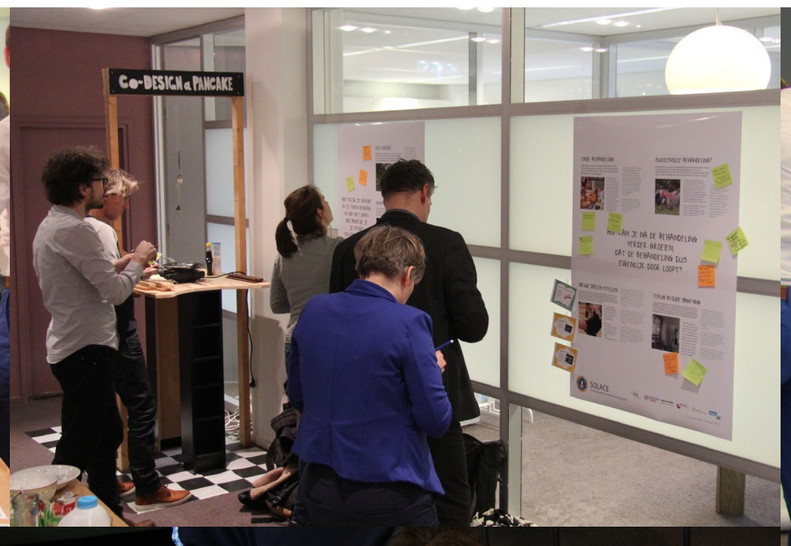

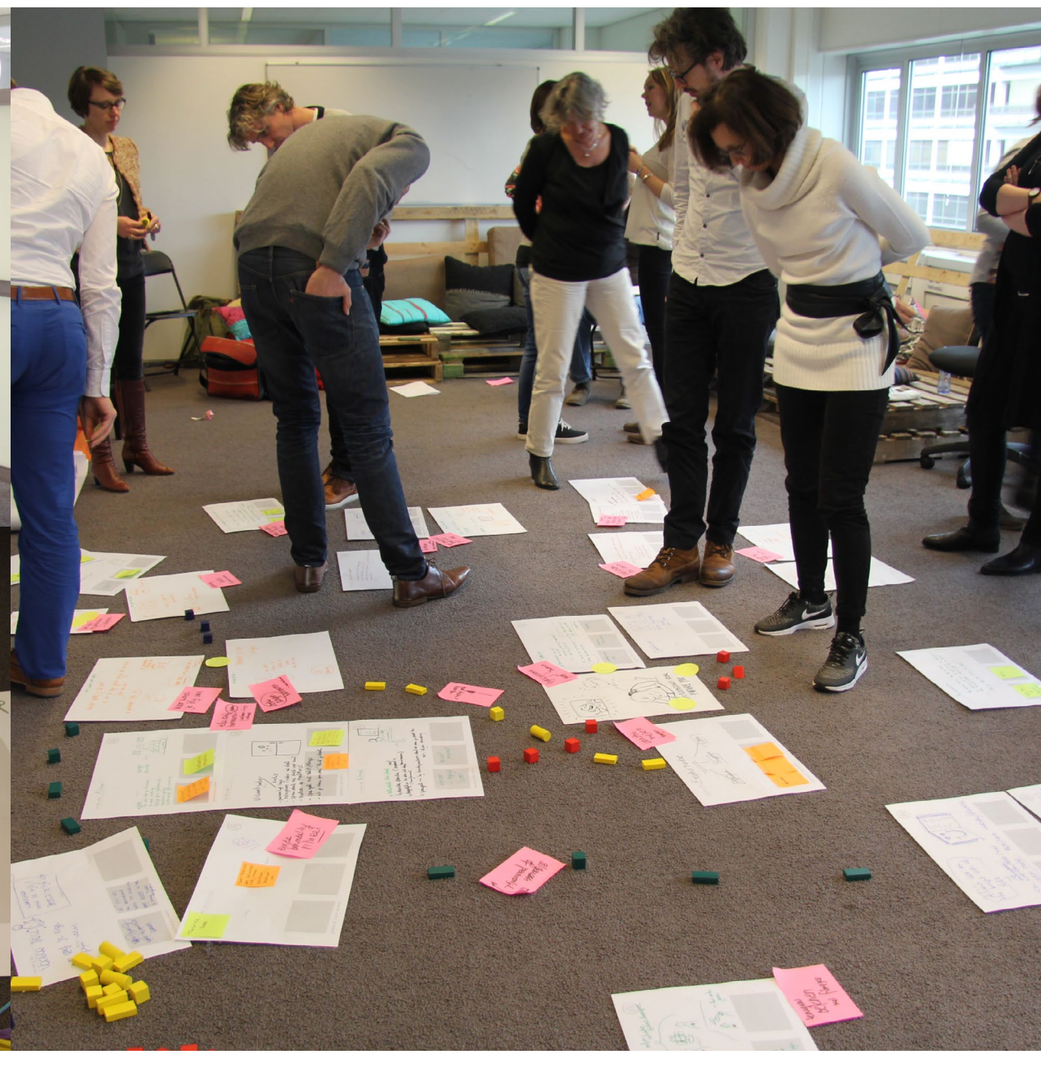
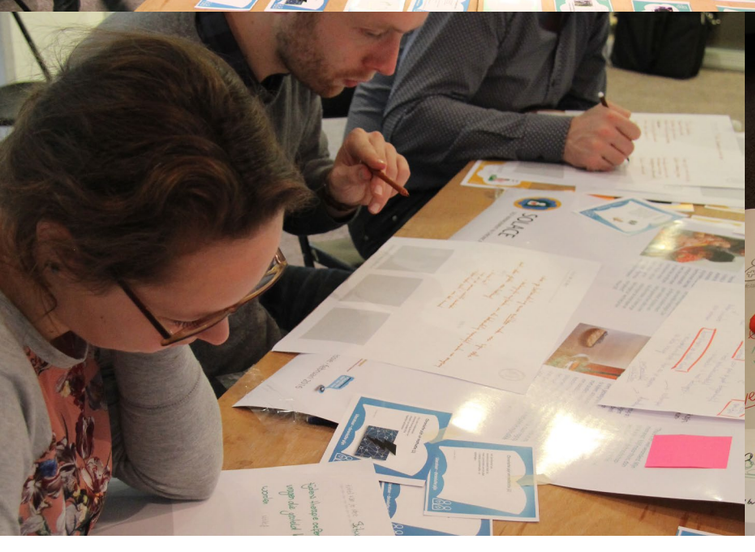
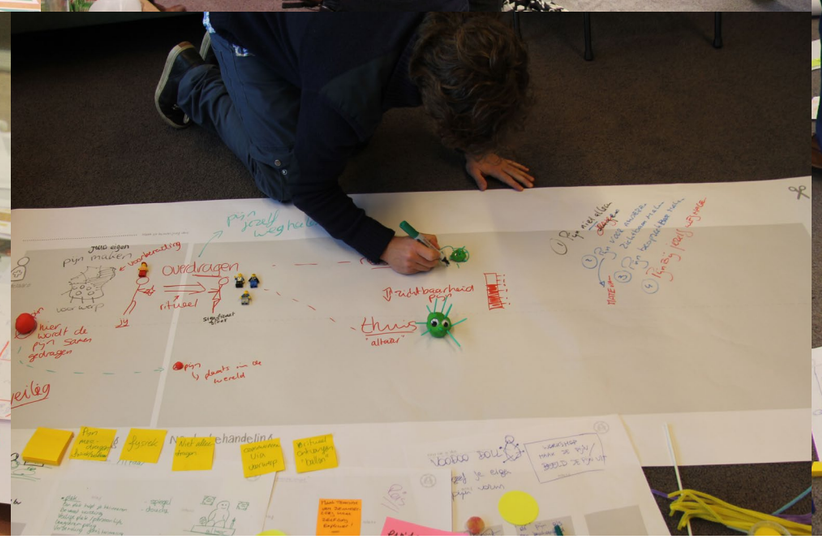

Overview of the co-creation session. By means of various assignments, stakeholders were encouraged to interact with each other and to use the data to develop intervention concepts.

Five concept ideas

Throughout the session, a professional draftsman visualized the emerging concept ideas. For example, 'time machine' included making videos to register progress and to allow 'time travel' trough earlier treatment experiences.

Time Machine

- Using videos to register progress and to allow 'time travel' after treatment.
- Helps to visualize progress and to remember important treatment experiences.

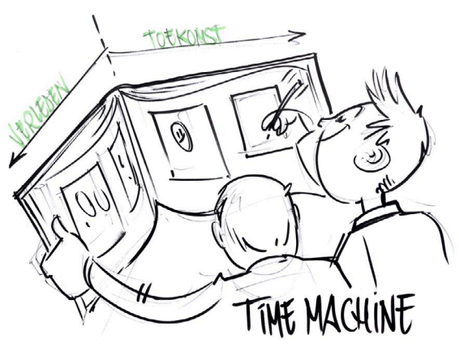

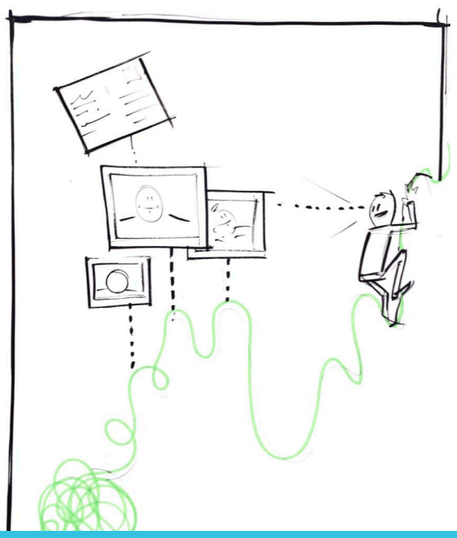
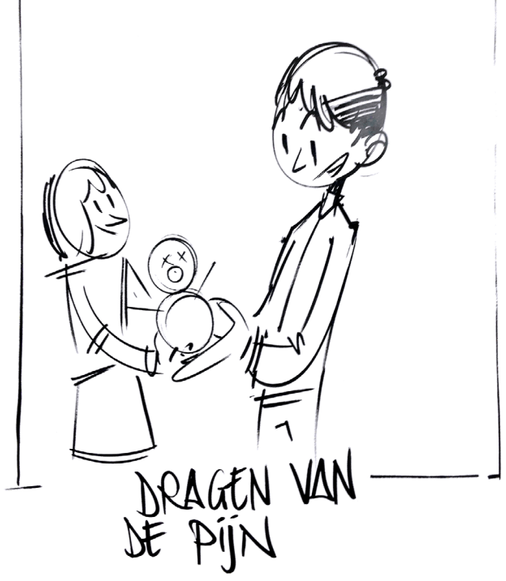

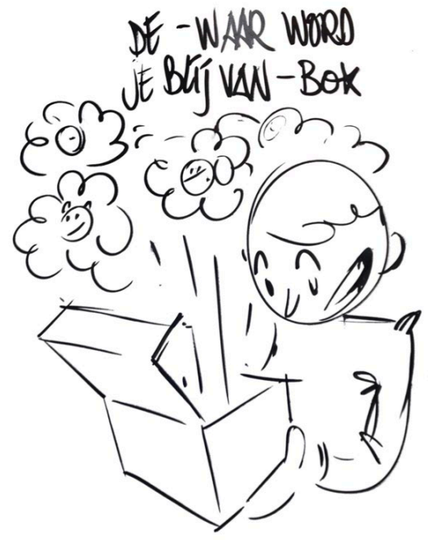

SOLACE

SELF MANAGEMENT IN CHRONIC PAIN STRATEGIES

Phase 3 'develop'

3.1 Concept development

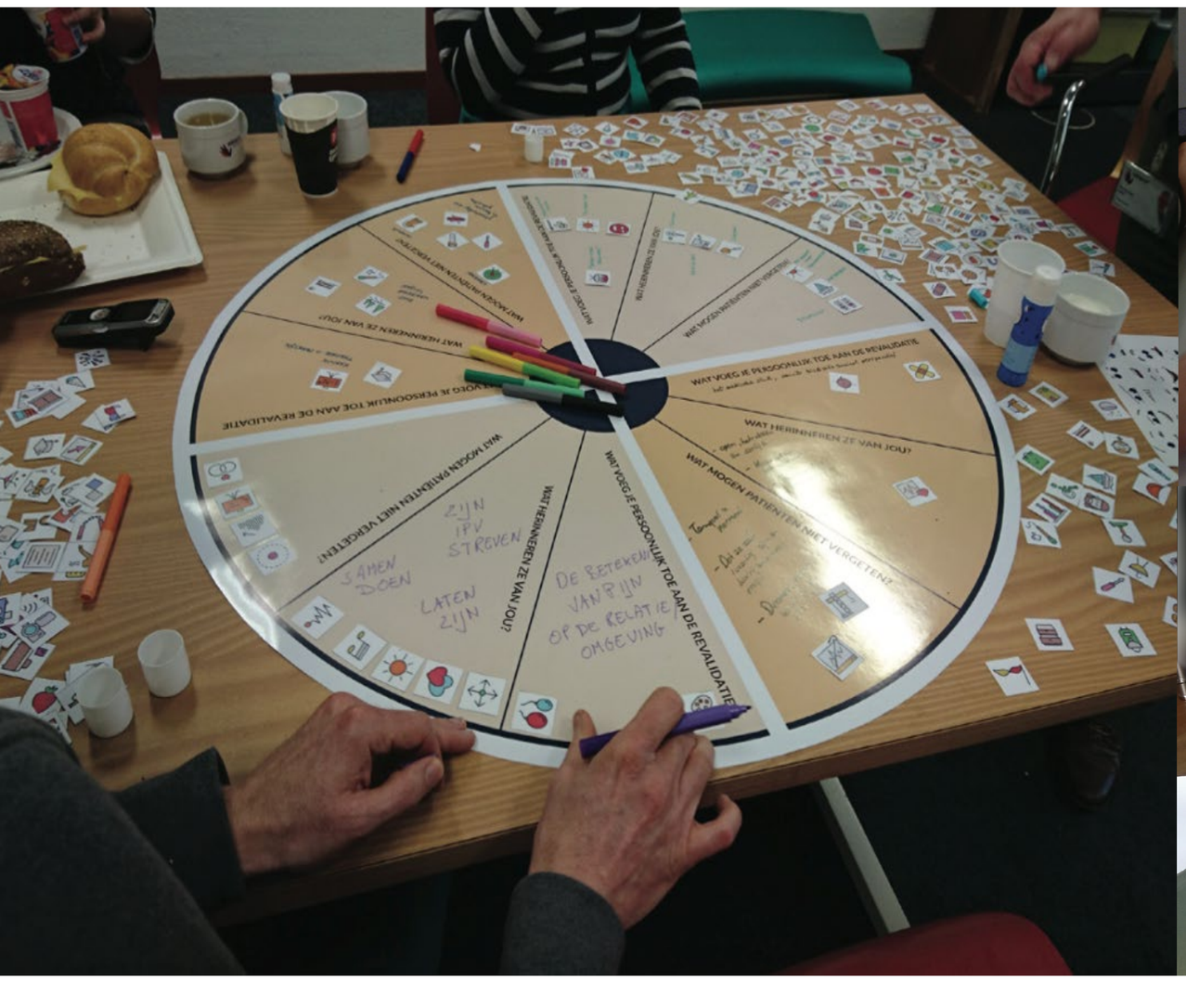

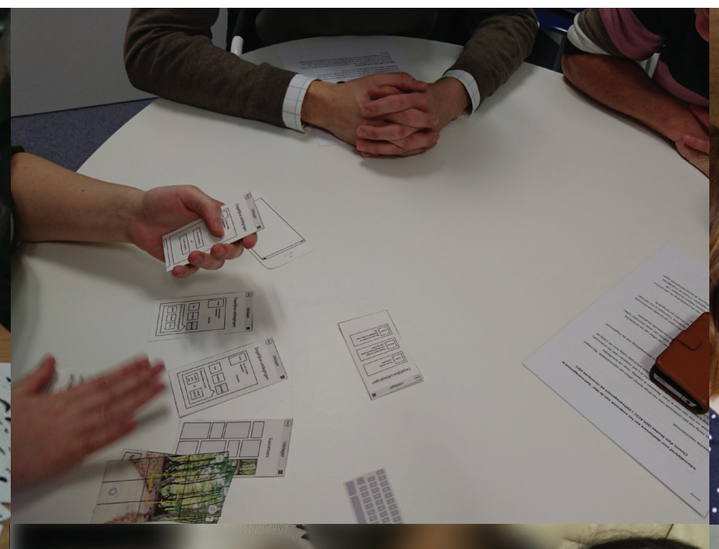

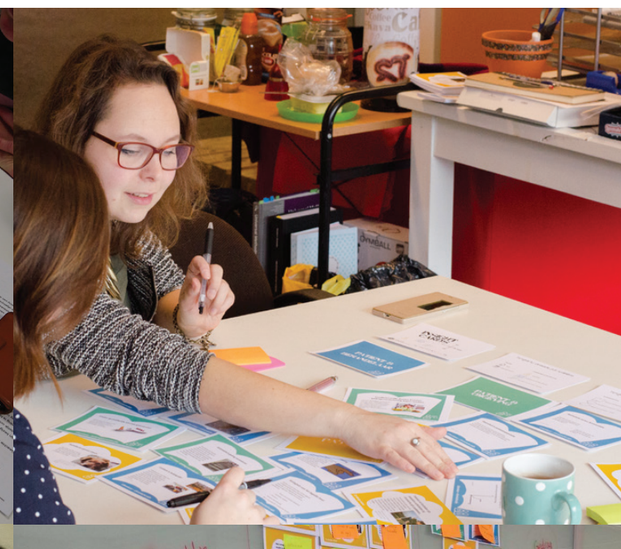

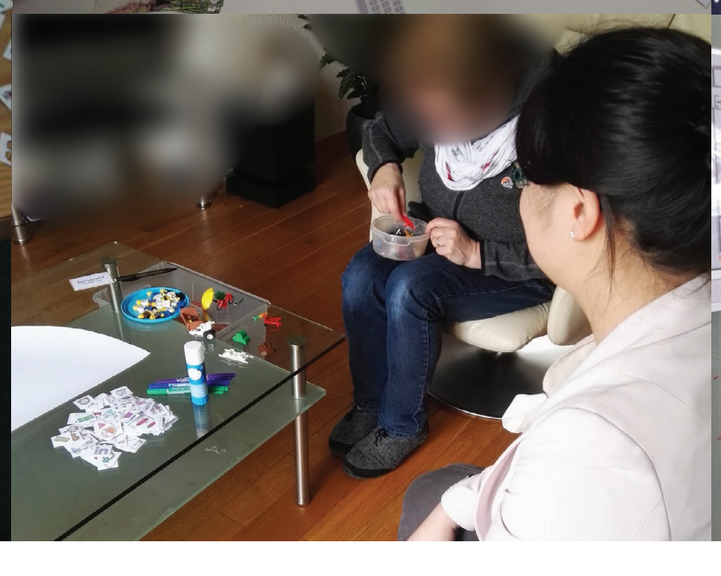

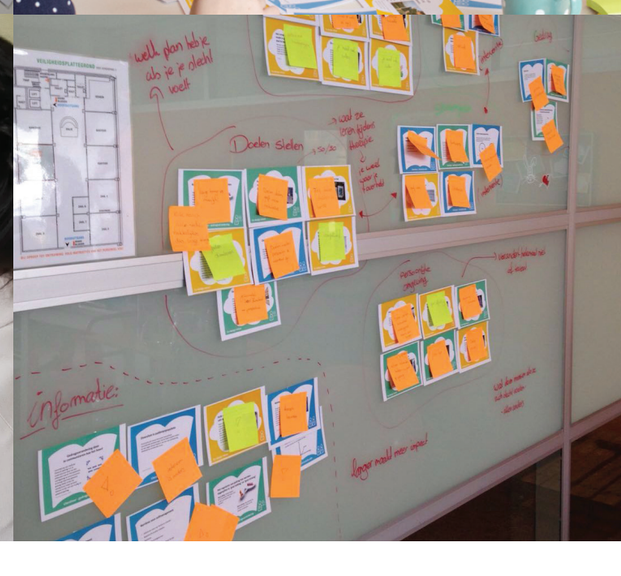

Pictures that were taken during various stakeholder and analysing sessions in the develop phase. Two student teams further developed the concepts into rudimentary prototypes. They regularly involved patients and health providers to test and refine their ideas.

Two rudimentary prototypes

The end result of the develop session: two rudimentary prototypes.

SOLAPP
MOBIELE APPLICATIE

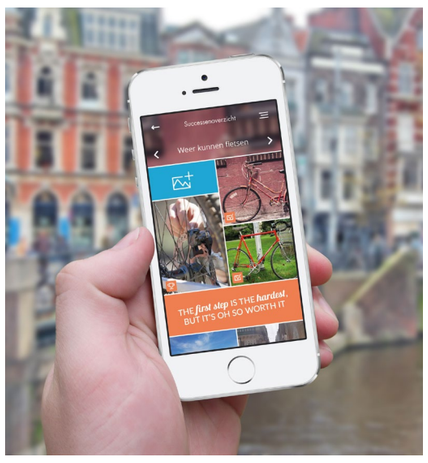

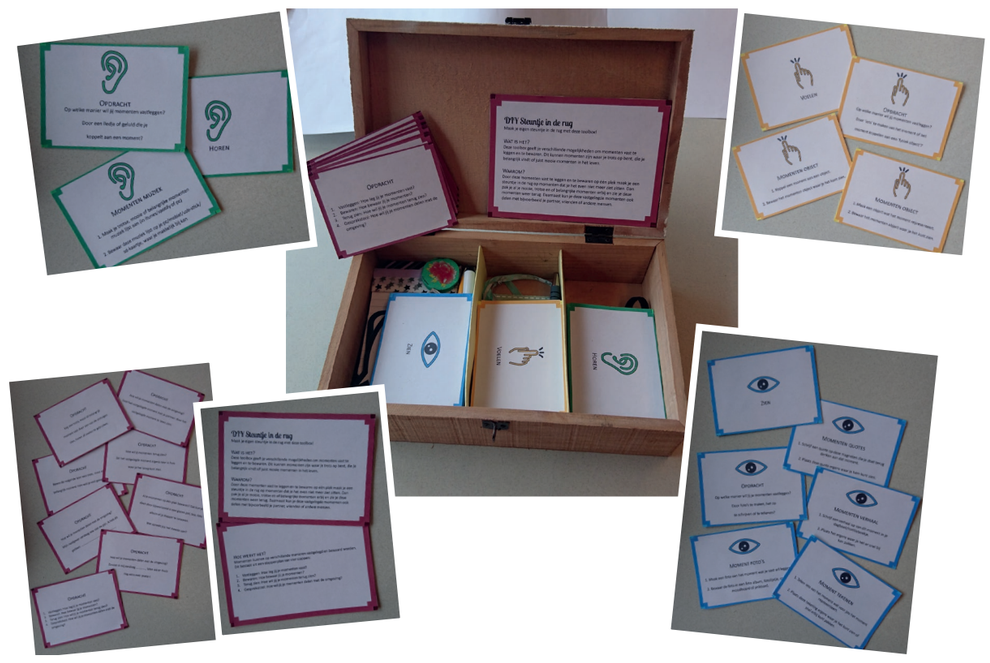

SOLACE
SELF MANAGEMENT IN CHRONIC PAIN STRATEGIES

Phase 3 'develop'

3.2 DIY Toolkit

The DIY toolkit contains various means to capture and store relevant treatment insights. These means are grouped by the corresponding type of storage: visually (e.g. a picture); by text (e.g. an associated quote); or by sound (e.g. a voice recording).

The toolkit also provides suggestions for how to store the insights after treatment and how to share this information with significant others or healthcare providers.

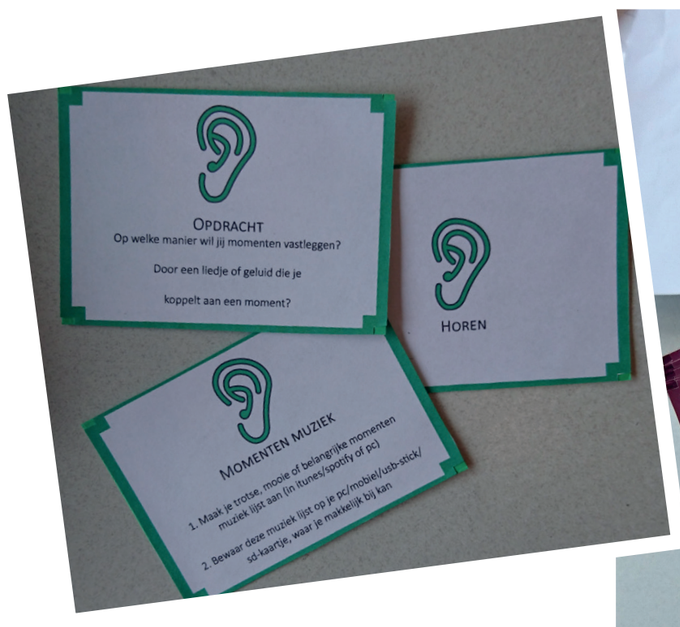

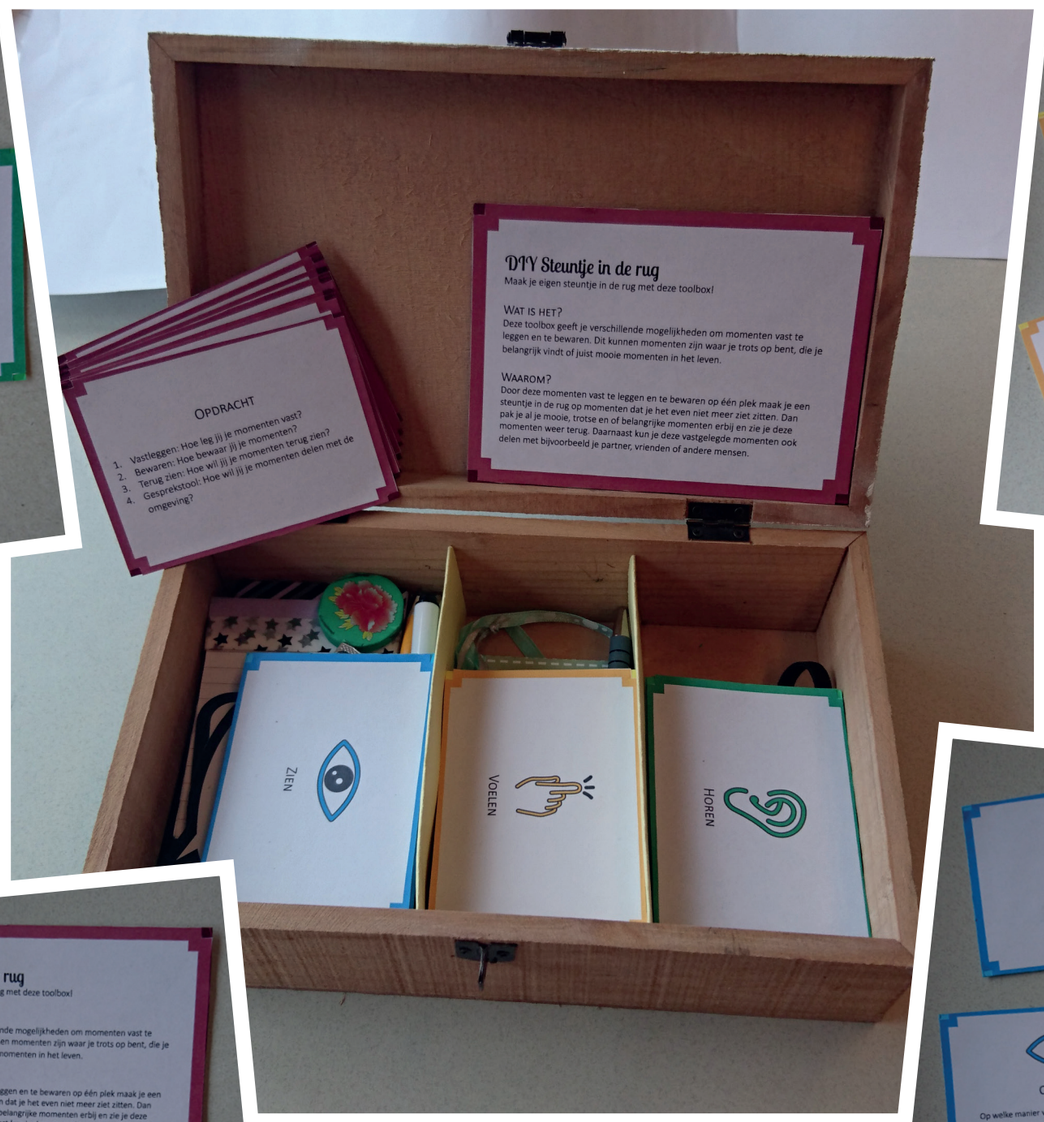

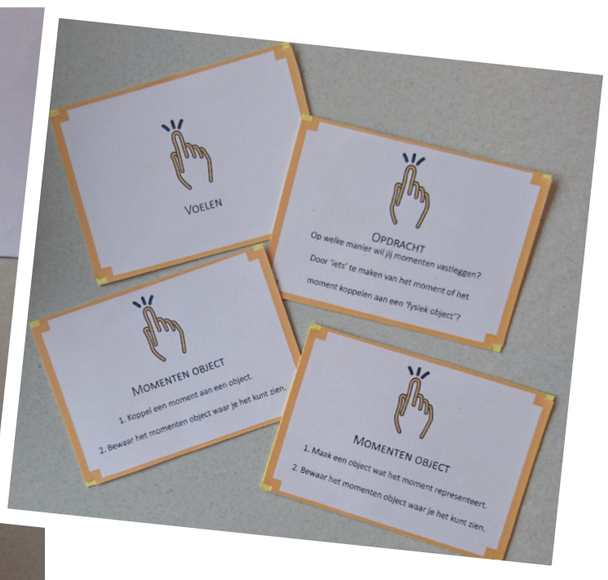

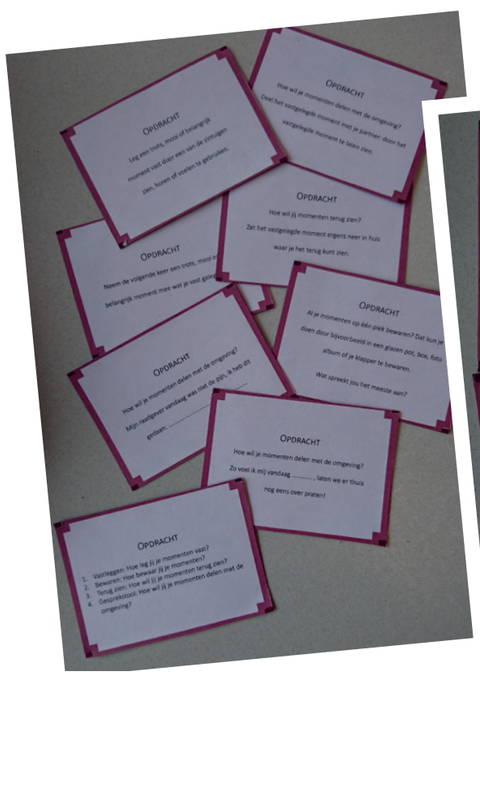

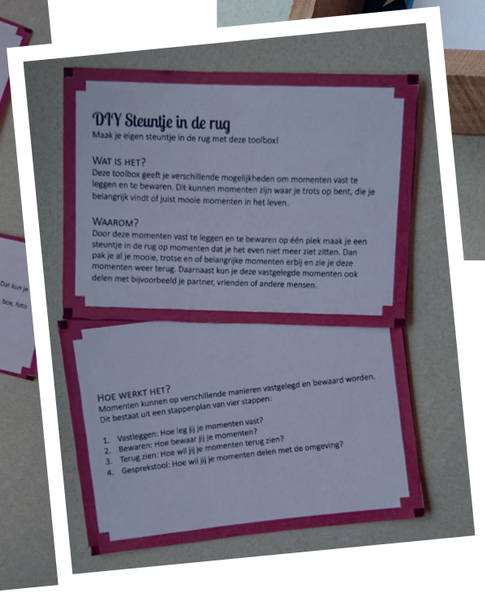

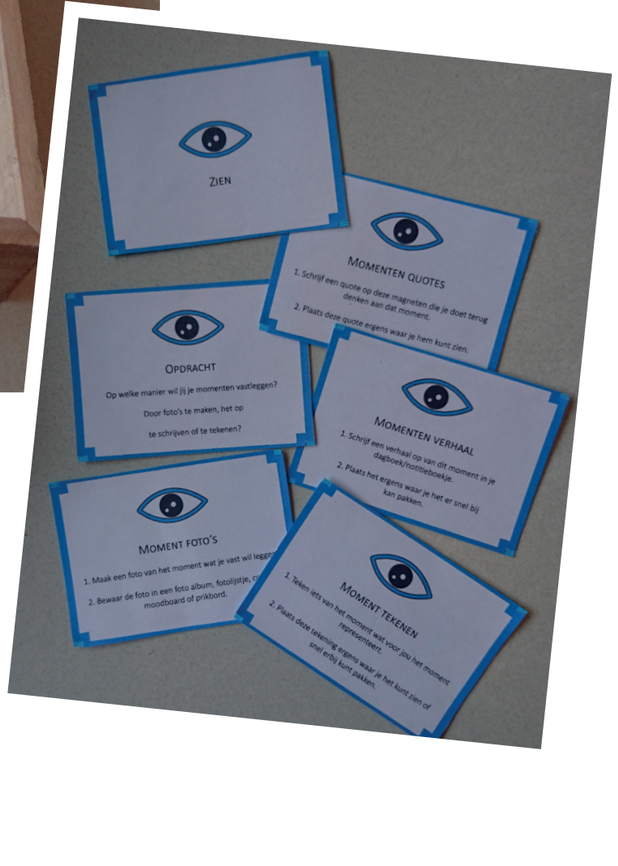

SOLACE

SELF MANAGEMENT IN CHRONIC PAIN STRATEGIES

Phase 3 ‘develop’

3.3 Solapp

The Solapp application provides patients with the opportunity to describe important personal values and subsequently formulate related personal goals. The application provides a structure where patients can gradually progress towards each goal by means of planning specific ‘steps’.

SOLAPP
MOBIELE APPLICATIE

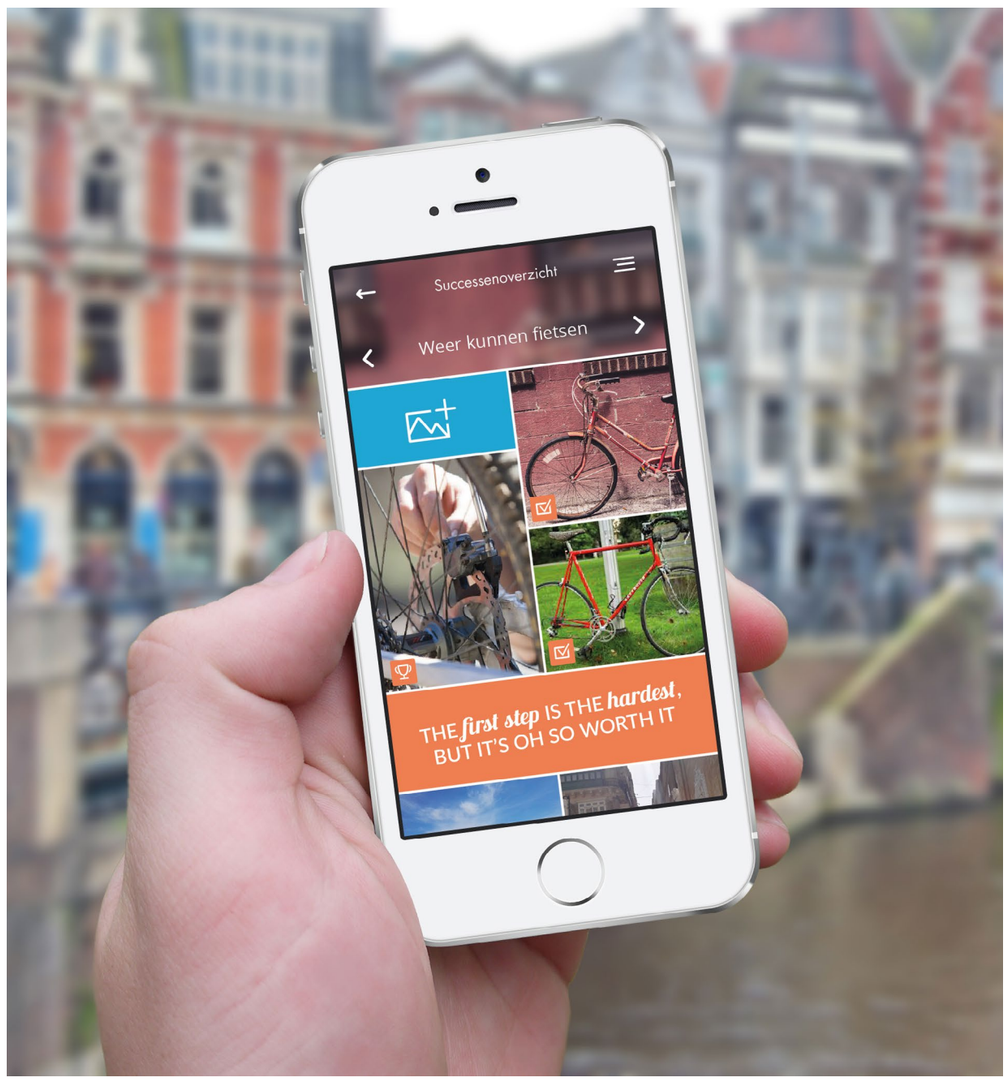

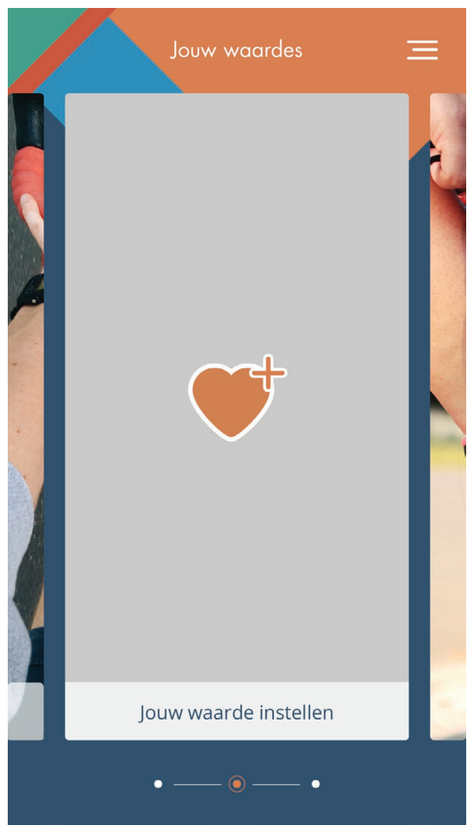

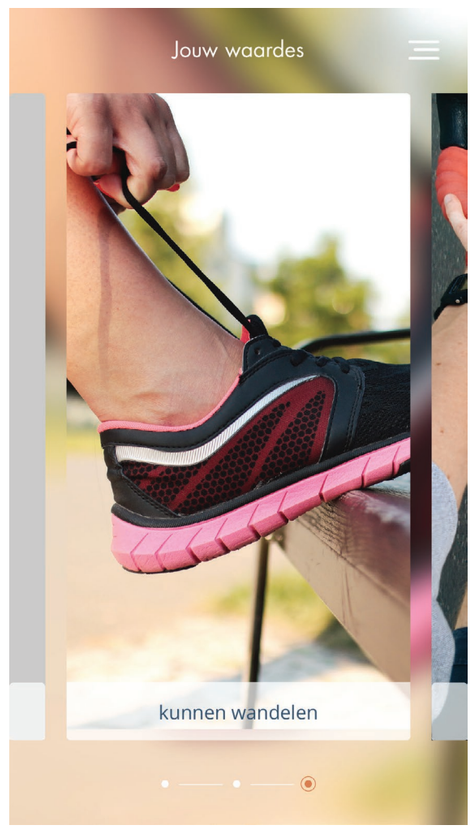

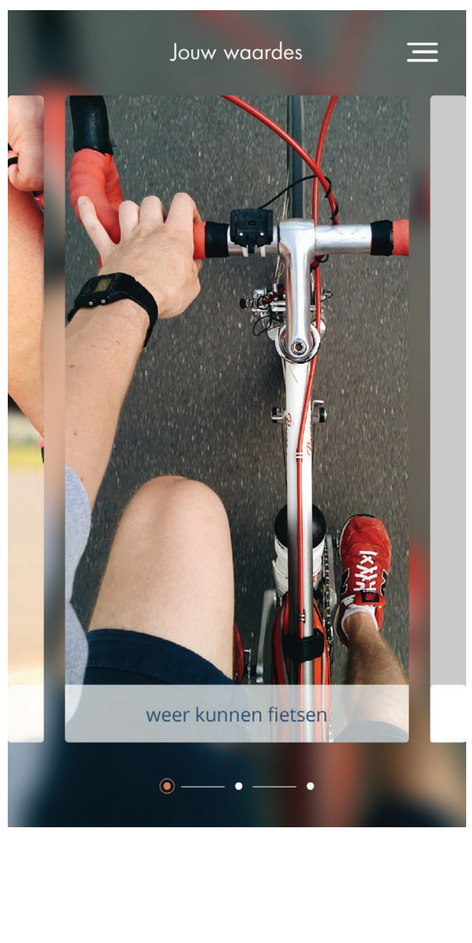

OVERZICHT WAARDES

- Inzicht 7 – Visueel maken
- Inzicht 16
- Uitspraak 47
- Iteratie pag. 90

- Ontwerpcriteria 2
- Ontwerpcriteria 4
- Ontwerpcriteria 5
- Ontwerpcriteria 11

- Inzichtkaart 06
- Inzichtkaart 07
- Inzichtkaart 09

Phase 4 ‘deliver’

4.1 Co-creation session 2

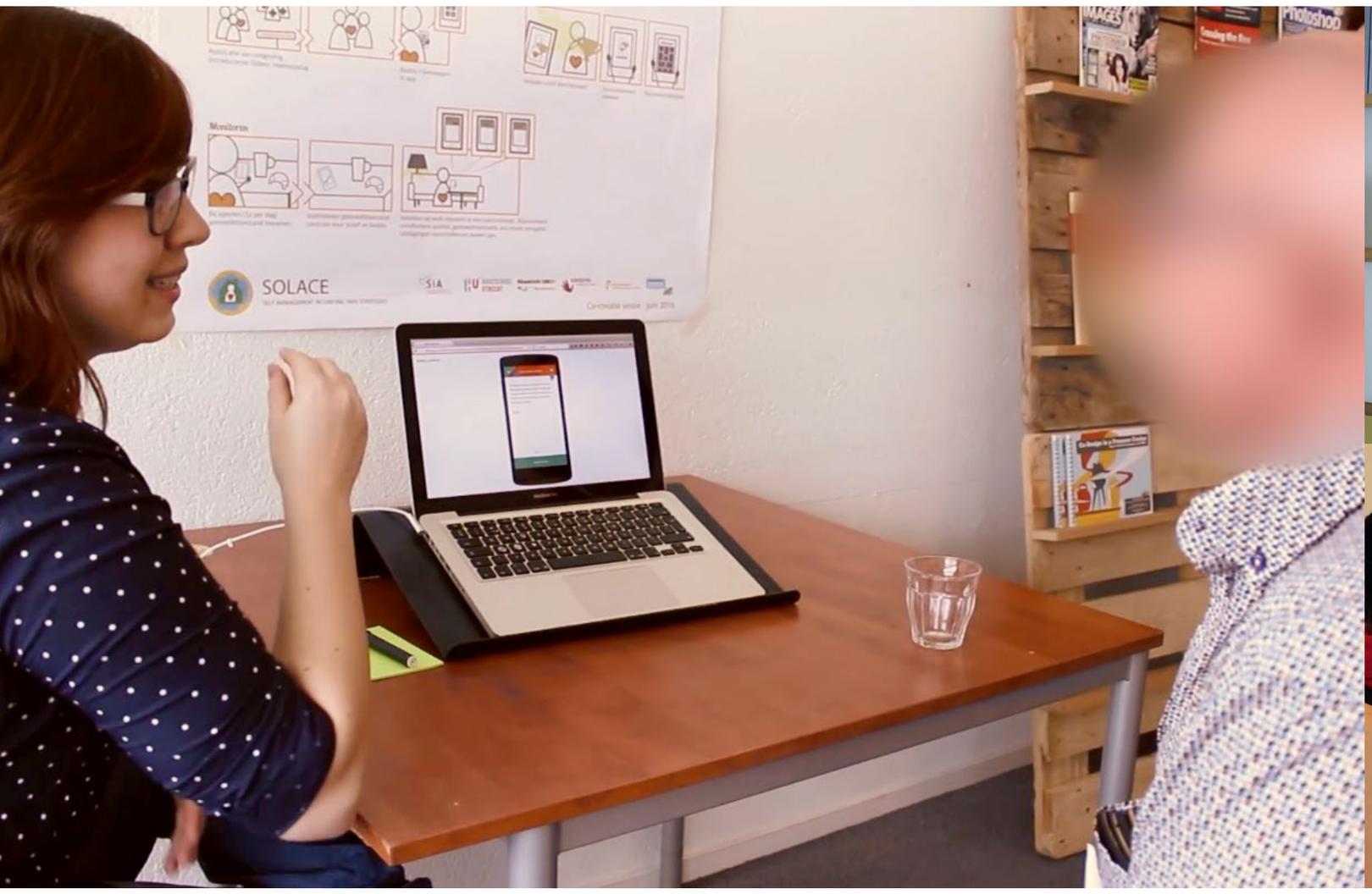

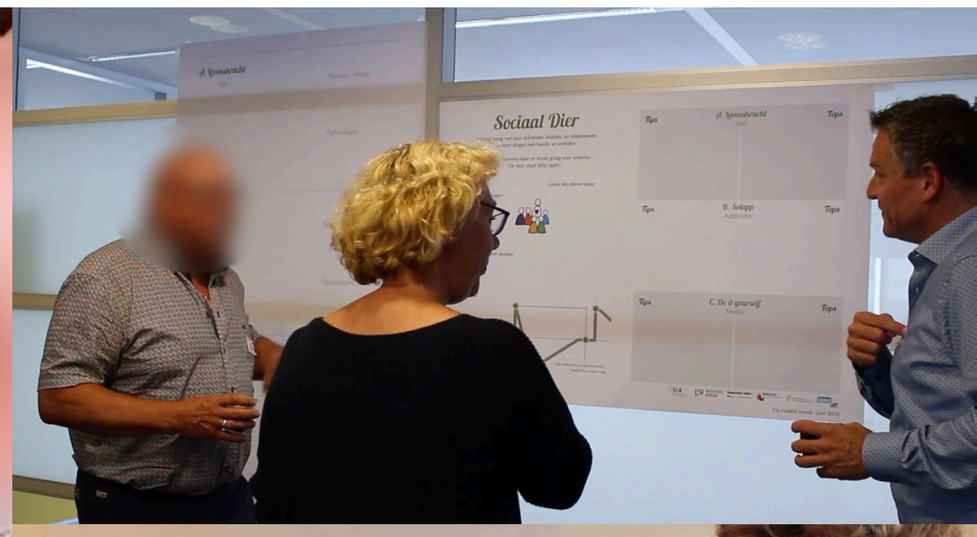

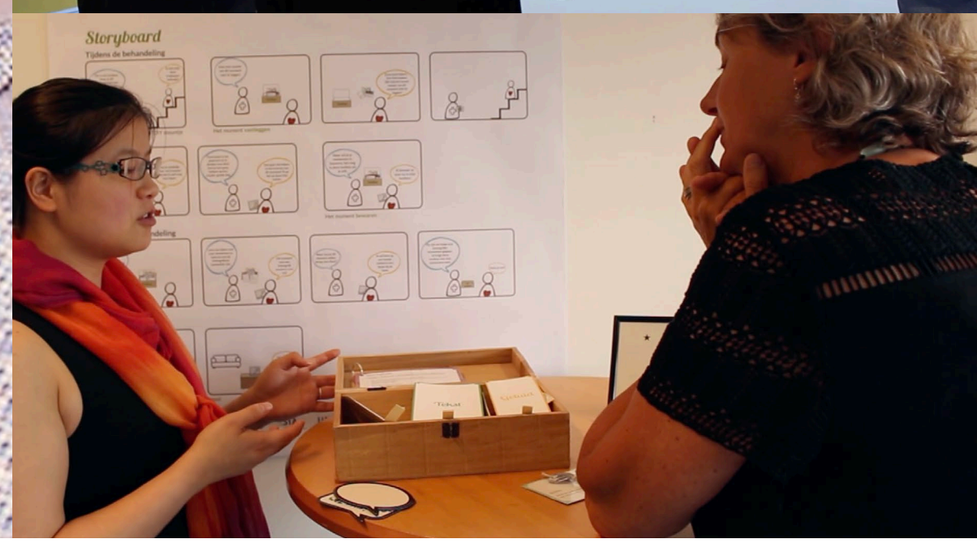

Overview of the co-creation session where students presented their prototypes and stakeholders discussed their use and function.

Personas

4 personas that were based on extremes two characteristics: extravert versus introvert and underuse versus overuse. This tool helped to simulate the adaptation of both prototypes for each of these four personas, providing an emphasis on the patient perspective.

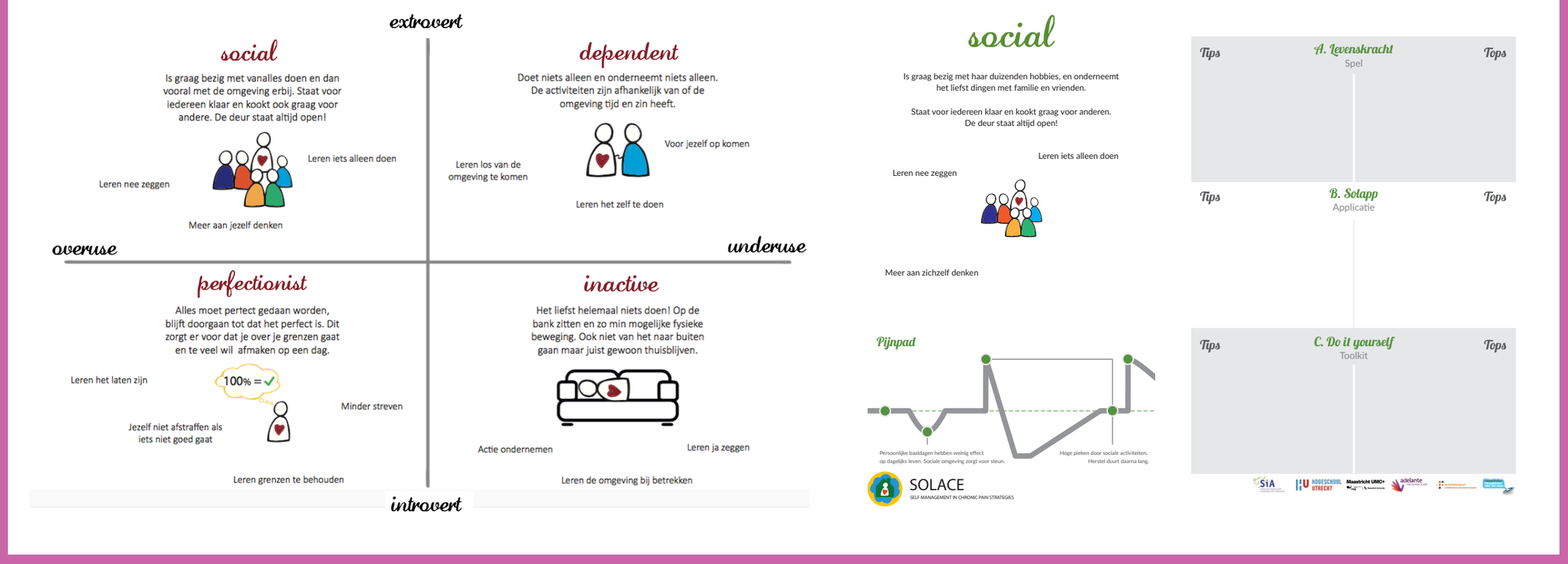

SOLACE

SELF MANAGEMENT IN CHRONIC PAIN STRATEGIES

Phase 4 ‘deliver’

4.2 Intervention components

Both interventions were divided into multiple separate components. For each component we evaluated to what extent it corresponded with (sub)themes from the qualitative dataset. Furthermore, we performed literature searches to find ways to improve each component.

For example:

Component ‘select personal values’

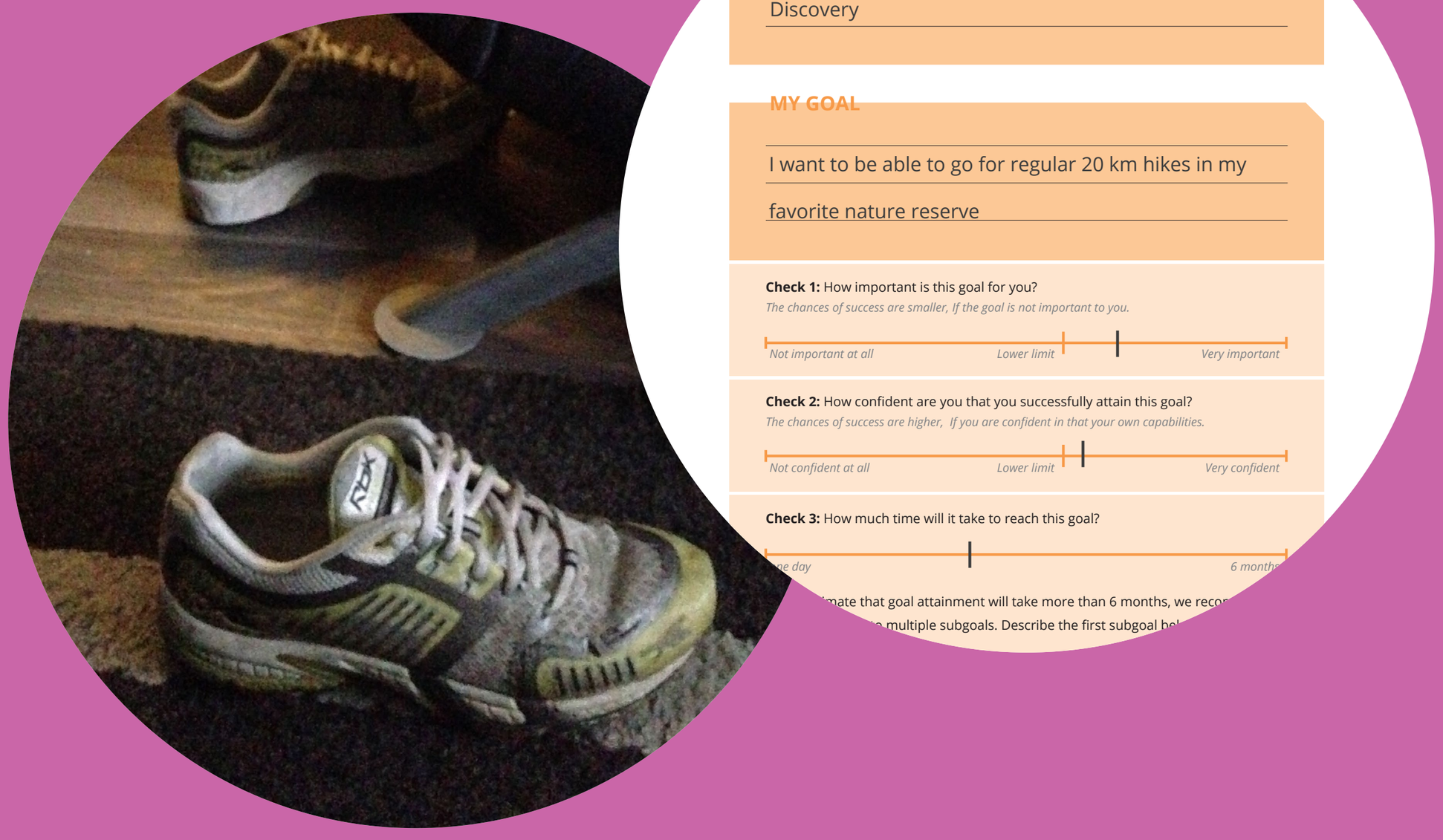

Theme 2_Goals and values
Subtheme 1_Remembering goals and values

“Walking was my most important goal. Why? Because it gives me freedom.”
- patient, 2.1

BCT 13.4_Valued self-identity

Chase et al (2013) Values are not just goals

SOLACE
SELF MANAGEMENT IN CHRONIC PAIN STRATEGIES

Phase 4 'deliver'

4.3 Prototype workbook

English translation of four pages of the workbook prototype:

a_front page

b/c_value based goal-setting procedure

d_example of two insight cards.

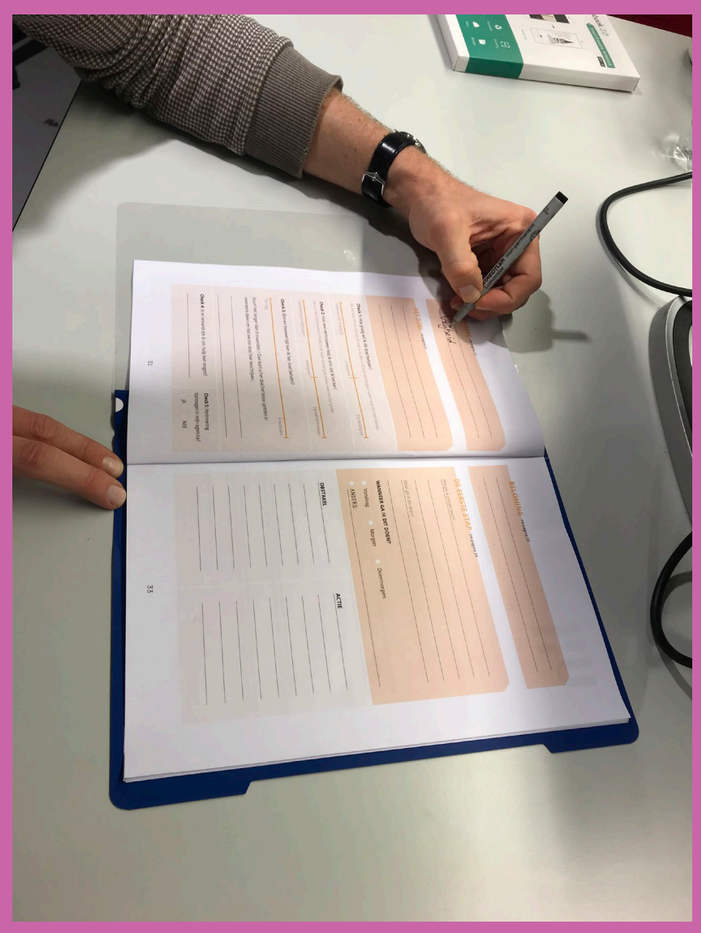

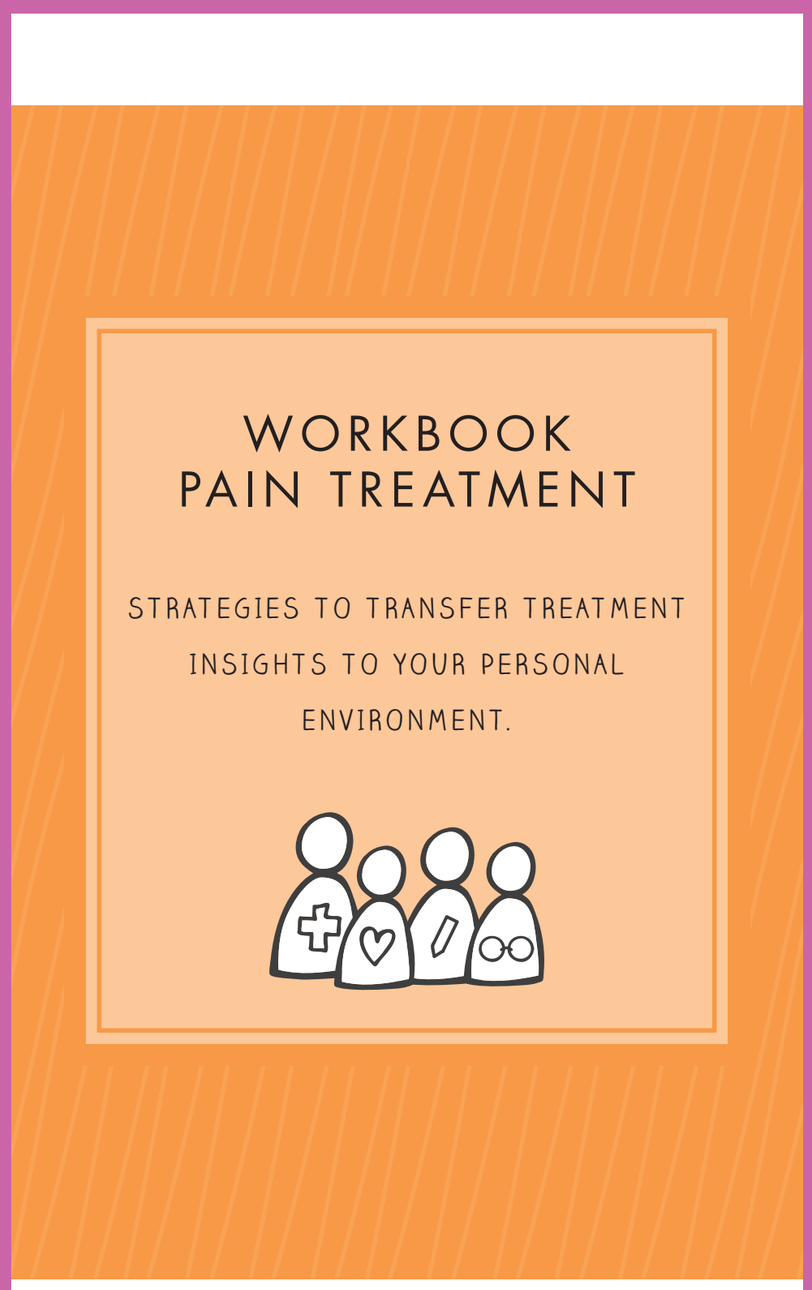

REWARD

Going for dinner after the first successful walk

THE FIRST STEP

what will I do?

Meet with Jane to plan training sessions and go for a short walk

Where will I do this? At my house

WHEN WILL I DO THIS?

○ today ○ tomorrow ○ day after

X ELSE: Monday, next week

OBSTACLE

Not in the mood, tired, having pain

Bad weather

ACTION

Apply pacing strategies during the day

Just go. Keep on paved roads

MY VALUE

Discovery

MY GOAL

I want to be able to go for regular 20 km hikes in my favorite nature reserve

Check 1: How important is this goal for you?

The chances of success are smaller, If the goal is not important to you.

Not important at all — *Lower limit* — *Very important*

Check 2: How confident are you that you successfully attain this goal?

The chances of success are higher, If you are confident in that your own capabilities.

Not confident at all — *Lower limit* — *Very confident*

Check 3: How much time will it take to reach this goal?

one day — *6 months*

If you estimate that goal attainment will take more than 6 months, we recommend to divide the goal into multiple subgoals. Describe the first subgoal below:

Check 4: Is there someone that you can ask for help?

my friend Jane

Check 5: Add a calender reminder?

X YES ○ NO

MOMENT

I HAD A PLEASANT INTERACTION WITH A NEW PERSON IN OUR GROUP. I SHARED MY EXPERIENCES AND ENCOURAGED HIM TO CONTINUE EXERCISING, IRRESPECTIVE OF PAIN OR TIREDNESS.

MEMORY CUE

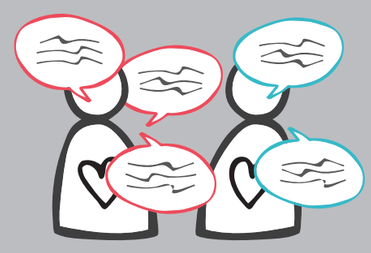

MOMENT IMPORTANT TOPIC DURING TODAY'S SESSION:

HURT DOES NOT EQUAL HARM.

MEMORY CUE

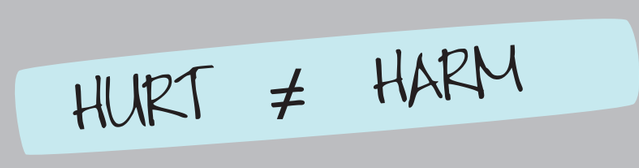

SOLACE

SELF MANAGEMENT IN CHRONIC PAIN STRATEGIES
